# Antihypertensive Activity of Milk Fermented by *Lactiplantibacillus* *plantarum* SR37-3 and SR61-2 in L-NAME-Induced Hypertensive Rats

**DOI:** 10.3390/foods11152332

**Published:** 2022-08-04

**Authors:** Lin Yuan, Ying Li, Moutong Chen, Liang Xue, Juan Wang, Yu Ding, Jumei Zhang, Shi Wu, Qinghua Ye, Shuhong Zhang, Runshi Yang, Hui Zhao, Lei Wu, Tingting Liang, Xinqiang Xie, Qingping Wu

**Affiliations:** 1 School of Food and Biological Engineering, Shaanxi University of Science and Technology, Xi’an 710021, China; 2 Guangdong Provincial Key Laboratory of Microbial Safety and Health, State Key Laboratory of Applied Microbiology Southern China, Institute of Microbiology, Guangdong Academy of Sciences, Guangzhou 510070, China

**Keywords:** *Lactiplantibacillus plantarum*, probiotic fermented milk, ACE, gut microbiota, hypertension

## Abstract

Probiotic fermented milk can lower the incidence rate of hypertension and is beneficial to the regulation of the intestinal microecology. However, the underlying molecular mechanism remains elusive. Here, we evaluated the role of the gut microbiota and its metabolites in the antihypertensive effect of milk fermented by the *Lactiplantibacillus plantarum* strains SR37-3 (PFM-SR37-3) and SR61-2 (PFM-SR61-2) in Ng-nitro-L-arginine methyl ester induced hypertensive rats. The results showed that PFM-SR37-3 and PFM-SR61-2 intervention significantly lowered the blood pressure (BP) of NG-nitro-L-arginine methyl ester induced hypertensive rats and attenuated renal injury. In particular, long-term administration of PFM inhibited a progressive elevation in SBP (170.22 ± 8.40 and 133.28 ± 6.09 by model group and PFM-SR37-3 treated model group, respectively, at the end of the 4 weeks; *p* < 0.01 PFM-SR37-3 treated model group versus model group) and DBP (133.83 ± 5.91 and 103.00 ± 6.41 by model group and PFM-SR37-3 treated model group, respectively, at the end of the 4 weeks; *p* < 0.01 PFM-SR37-3 treated model group versus model group). PFM-SR37-3 and PFM-SR61-2 reshaped the gut microbiome and metabolome, and especially regulated the metabolic levels of L-phenylalanine, L-methionine and L-valine in the intestine and blood circulation. The analysis of the target organ’s aortic transcriptome indicated that the protective effects of PFM-SR37-3 and PFM-SR61-2 were accompanied by the modulation of the BP circadian rhythm pathway, which was conducive to cardiovascular function. Vascular transcriptomic analysis showed that circadian rhythm and AMPK might be potential targets of hypertension. In addition, the ACE inhibition rates of *Lactiplantibacillus plantarum* SR37-3 and *Lactiplantibacillus plantarum* SR61-2 in vitro were 70.5% and 68.9%, respectively. Our research provides new insights into novel and safe options for hypertension treatment.

## 1. Introduction

Hypertension is the leading cause and risk factor of cardiovascular disease (CVD). In addition to kidney and cerebrovascular injury [1], it is also a well-defined risk factor for coronary heart disease, stroke and atherosclerosis [2]. The management of hypertension often entails long-term treatment based on a combination of drugs, but studies have shown that antihypertensive drugs can be enriched in the body and initiate side effects. Therefore, there is an urgent need for new methods to prevent and control this disease. There is a growing line of literature research that is in favor of the health benefits attributed to probiotic and their fermented milks such as a reduction in the serum cholesterol level, the prevention of various types of cancer, hypoglycemic effects, the modulation of brain activity, the improvement of cognitive impairments, the improvement of gut health, the enhancement of immunity and blood pressure (BP) lowering [3,4,5,6,7]. Meanwhile, the side-effects of probiotics-fermented milk (PFM) intervention are also rarely reported. The applications of BP-lowering probiotics in food are mainly focused on dairy products. According to research, probiotics such as lactic acid bacteria can hydrolyze the protein in dairy products to produce some short peptides, which have the effect of lowering BP. In particular, two short peptides, Ile-Pro-Pro (IPP) and Val-Pro-Pro (VPP), are as effective as antihypertensive drugs in treating hypertension (clinical practice). In addition to milk proteins as a source of bioactive peptides, ten Ace-inhibiting peptides were also identified from dromedary milk produced by *Lactobacillus helveticus* or *Lactobacillus acidophilus*, which have good stability and can be later recovered and added to food to make food products with ACE inhibitory activity [8]. Further studies revealed that these two short peptides, IPP and VPP, may take effect by inhibiting the activity of Angiotensin Converting Enzymes (ACE).

With the development of sequencing technologies, gut microbiota are constantly mined. Studies have shown that the human gut maintains a stable state. If this homeostatic state is disturbed by certain factors, it will be destroyed, resulting in the occurrence of diseases [9]. The dysbiosis of gut microbiota can mainly reduce microbial diversity and richness [10]. Nine randomized controlled trials (RCTs) were subjected to a meta-analysis. The results showed that, compared with the control group, the systolic BP (SBP) of the experimental group decreased by 3.56 (95%CI−6.46~−0.66) mmHg and the diastolic BP (DBP) decreased by 2.38 (95%CI−2.38~−0.93) mmHg after taking probiotics [11]. Researchers have been exploring the mechanism of BP reduction after taking probiotic preparations. Probiotics can use their metabolites to regulate BP. For example, the intestinal metabolite, SCFA, is activated by the receptor to regulate the secretion of renin to achieve the regulation of BP. The specific mechanism is that short-chain fatty acids activate Gpr41 and Olfr78 receptors, which regulate BP with vasodilator and angiotensin [12].

In addition, hypertension is associated with inflammation and autoimmunity. The oxidative stress and inflammatory infiltration of the renal interstitium and vascular wall can lead to an elevated BP [13]. When stimulated to raise the BP, cells produce a great number of inflammatory cytokines [14], which cause vascular endothelial dysfunction and vascular resistance to increase. Masson et al. found that in spontaneously hypertensive rats (SHR), tumor necrosis factor α (TNF-α), interleukin-6 (IL-6) protein expression and the phosphorylation of the human nuclear factor inhibitory protein α increased, in addition to increasing the mean arterial pressure, heart rate and vasomotor sympathetic activity [15].

Accumulating evidence indicates that there is a close relationship between gut microbiota dysbiosis and the development of hypertension. Therefore, studying the relationship between probiotics and hypertension, exploring the exact mechanism of probiotics to reduce BP and developing probiotic products with the function of reducing BP reduction function can help solve the side effects experienced by hypertensive patients who cannot be cured and that are caused by lifelong medication. Except for probiotic-fermented dairy products that produce antihypertensive peptides, the regulation of probiotics or probiotic fermented food on the gut microbiota may be an alternative choice for controlling BP. Whether it is the dysregulation of gut microbiota that leads to the development of hypertension or that hypertension leads to the dysregulation of gut microbiota, the specific mechanism requires further research. This paper mainly focused on the antihypertensive effect of two *Lactiplantibacillus plantarum* strains in vitro and in vivo, and explored their antihypertensive mechanism. The reason why *Lactiplantibacillus plantarum* SR37-3 and SR61-2 was chosen was that it was reported that *Lactiplantibacillus* plantarum could lower BP [16,17].

## 2. Materials and Methods

### 2.1. Animals and Study Design

In total, 24 specific pathogen-free (SPF) Wistar male rats (8 weeks of age) were obtained from Southern Medical University in China (Guangdong, China). The rats were placed in a controlled environment (a temperature of 23 ± 3 °C, a relative humidity of 50–60% and a light/dark cycle of 12/12 h), and free water and food were provided during the experiment. The rats were housed individually and acclimated to the new environment for approximately 7 days. The rats were then randomized in four groups (*n* = 6 per group), and each group had the following characteristics: (1) animals who were not treated with N^G^-nitro-L-arginine methyl ester (L-NAME) received standard chow (W), (2) animals treated with L-NAME who received standard chow (LN), (3) L-NAME-treated rats who were administered 10 mL/kg milk fermented by the *Lactiplantibacillus plantarum* strain SR37-3 (PFM-SR37-3) daily for 4 weeks via the oral gavage route (LN + SR37-3), and (4) L-NAME-treated rats who were administered 10 mL/kg milk fermented by the *Lactiplantibacillus plantarum* strain SR61-2 (PFM-SR61-2) daily for 4 weeks via the oral gavage route (LN + SR61-2). *Lactiplantibacillus plantarum* strain SR37-3 and *Lactiplantibacillus plantarum* SR61-2 are from salted fish. The biological deposit information of *Lactiplantibacillus plantarum* strain SR37-3 and *Lactiplantibacillus plantarum* strain SR61-2 are GDMCC No: 62390 and GDMCC No: 62389, respectively. The experimental design was approved by the Laboratory Animal Management and Ethics Committee of the Guangdong Institute of Microbiology (GT-IACUC202010301) and followed the standard guidelines for maintenance. At the end of the experiment, the rats were anesthetized with a subcutaneous injection of 30 mg/kg Zoletil 50 (Virbac Co., Ltd., Carros, France), and blood was collected from the heart. After the rats were sacrificed, the kidney, thoracic aorta, colon and cecum were removed immediately, frozen with dry ice and stored at temperature −80 °C.

### 2.2. L-NAME Model

The hypertensive state of rats was achieved by adding the NOS inhibitor L-NAME at a concentration of 400 mg/L in drinking water [18].

### 2.3. Diets/Treatment

The Co60 irradiation maintenance diet (SWS9102, Xietong, Jiangsu, China) contains ≥50 g/kg of crude fiber, ≥40 g/kg of crude fat, ≤80 g/kg of ash, 6–12 g/kg of phosphorus, 10–18 g/kg of calcium, ≥180 g/kg of protein, ≥8.2 g/kg of lysine, ≥5.3 g/kg of methionine + cysteine and ≤100 g/kg of moisture. It is a standard balanced feeding pellet diet for rat feeding. This diet should keep them healthy, while eliminating the diet’s effects on blood pressure and blood chemistry. A total of 11% (*w*/*v*) skimmed milk from cow (D8340, Solarbio, Beijing, China) was prepared and sterilized at 105 °C for 15 min. A total 4 vol % each of the *Lactiplantibacillus plantarum* strains SR37-3 and SR61-2 was inoculated into sterilized skimmed milk respectively, cultured at 37 °C for 48 h and stored at −4 °C.

### 2.4. BP Measurement

Following the manufacturer’s instructions, we measured the BP using the tail-cuff method (BP-2010A System, Softron, Beijing, China). SBP and DBP were measured at baseline and after 2 and 4 weeks of intervention/treatment. The rats were acclimated before BP measurement. BP measurements were repeated three consecutive times for each rat to obtain the average value. If the animal showed an accelerated heart rate, indicating stress, then the BP was measured repeatedly on the same day. This method is improved on the basis of the research of Jie et al. [19].

### 2.5. Histological Analyses

The kidney was rinsed with PBS and fixed in 10% buffered formalin for 24 h. The kidney tissue was then dehydrated with an alcohol gradient, embedded in paraffin and sliced. Finally, the tissue was dewaxed using xylene and stained using hematoxylin-eosin (HE) [20]. We chose the kidney as the pathological section to know the protective effect of *Lactiplantibacillus plantarum* fermented milk on kidney damage caused by hypertension, because hypertension often causes some complications, especially the kidney [21,22].

### 2.6. Serum Biochemical Index Analysis

Blood was collected by heart puncture after fasting for about 16 h in order to conduct the serum biochemical analysis. The concentrations of biological indexes related to hypertension were determined by the commercial enzyme linked immunosorbent assay (ELISA) kits from Beijing Dongge Biotechnology Co., Ltd. (Beijing, China) following the manufacturer’s protocols [23].

### 2.7. Intestinal Microbial Diversity

#### 2.7.1. DNA Extraction and Polymerase Chain Reaction (PCR) Amplification

The microbial community’s genomic DNA was extracted from rat feces samples using the E.Z.N.A.^®^ Soil DNA Kit (Omega Bio-tek, Norcross, GA, USA) according to the manufacturer’s instructions. The DNA concentration and purity were determined using a NanoDrop 2000 UV-vis spectrophotometer (Thermo Scientific, Wilmington, NC, USA). The hypervariable regions V3–V4 of the bacterial *16S rRNA* gene were amplified with the primer pairs 338F (5’-ACTCCTACGGGAGGCAGCAG-3’) and 806R(5’-GGACTACHVGGGTWTCTAAT-3’) by an ABI GeneAmp^®^ 9700 PCR thermocycler (Applied Biosystems, Waltham, MA, USA). The PCR amplification of the *16S rRNA* gene was performed as follows: 3 min at 95 °C, followed by 25 cycles of 30 s at 95 °C, 30 s at 55 °C, 45 s at 72 °C and 10 min at 72 °C, and finally at 10 °C. The PCR mixtures contain 4 μL 5 × TransStart FastPfu buffer, 2 μL 2.5 mM dNTPs, 0.8 μL forward primer (5 μM), 0.8 μL reverse primer (5 μM), 0.4 μ TransStart FastPfu DNA Polymerase L, 10 ng template DNA and finally up to 20 μL ddH2O. PCR reactions were performed in triplicate. The PCR product was extracted and purified and quantified using a Quantus™ Fluorometer (Promega, Durham, NC, USA) according to the manufacturer’s instructions. All PCR amplifications were repeated three times [24].

#### 2.7.2. Illumina MiSeq Sequencing

Purified amplicons were pooled in an equimolar solution and paired-end sequenced on an Illumina NovaSeq PE250 platform (Illumina, San Diego, CA, USA) according to the standard protocols by Majorbio Bio-Pharm Technology Co. Ltd. (Shanghai, China) [24].

#### 2.7.3. Sequencing Data Processing

The raw *16S rRNA* gene sequencing reads were demultiplexed and quality-filtered by fastp version 0.20.0 [25] and merged by FLASH version 1.2.7. Operational taxonomic units (OTUs) with 97% similarity cutoff [26,27] were clustered using UPARSE version 7.1 [26] and chimeric sequences were identified and removed. The taxonomy of each OTU representative sequence was analyzed by RDP Classifier version 2.2 [28] against the *16S rRNA* database using a confidence threshold of 0.7.

### 2.8. Serum Metabolic Profile and the Metabolomic Determination of Gut Microbiota

We took 200 mL serum samples in a 1.5 mL EP tubes, extracted with 1 mL methanol-acetonitrile (V methanol: V acetonitrile = 1:1), and added with L-2-chlorophenylalanine (1 ng/mL) as the internal standard. After mixing well, the tube was placed in a 0 °C water ultrasonic processor for 20 min and transferred immediately to −20 °C for 1 h. The supernatants (approximately 1 mL) were transferred to 1.5 mL EP tubes after centrifuging with 13,000 rpm for 15 min at 4 °C. The extract was then dried in a vacuum concentrator without heating at 37 °C for about 8 h followed by the addition of 200 μL water–acetonitrile (V water: V acetonitrile = 1:1) into dried metabolites. The supernatants (approximately 180 μL) were transferred to a LC-MS glass vial after centrifuging with 13,000 rpm for 15 min at 4 °C. An equal volume of 2.5 μL was taken from each sample into the LC-MS vial as a quality control.

We weighed (50 ± 5) mg of solid stool sample, added 250 μL of precooled mixture (methanol: acetonitrile: water = 4:4:2, V:V:V) and homogenized it twice. It underwent ultrasonic cleaning in an ice bath for 10 min, and we let it stand at −20 °C for 1 h, and centrifuged it (12,000 rpm, 10 min, 4 °C) to take the supernatant. After drying at room temperature in a vacuum drying oven, we redissolved it with a 150 uL mixture (acetonitrile: water = 1:1, V:V), vortexed it for 30 s and centrifuged it to take the supernatant. We took 5 μL from each tube as the quality control sample.

The LC-MS system utilized an ACQUITY UPLC HSS T3 column (2.1 × 100 1.8 μm). We eluted it according to the gradient, the flow rate was 0.3 mL/min and the column temperature was 40 °C. The heating electrospray ion source (HESI) was adopted. The positive voltage was 3500 V and the negative voltage was 2000 V. The capillary temperature was 320 °C. The sheath gas flow rate was 45 arb and the auxiliary gas flow rate was 8 arb or 10 arb. We adopted the positive and negative ion switching acquisition mode. The Scan mode used was Full Scan/dd-MS2. The full MS resolution was set to MS Full Scan 70000FWHM and MS/MS17500FWHM. The metabolites were analyzed using SIMCA software (V14.1, MKS Data Analytics Solutions, Umea, Sweden) for principal component analysis (PCA) and orthogonal least squares discriminant analysis (OPLS-DA). R2 and Q2 values were used to describe the reliability of the data model.

### 2.9. RNA-Seq and Genome-Wide Transcriptome Analysis of Blood Vessels

#### 2.9.1. Sample Collection and Preparation

RNA from the thoracic aortas of rats and mRNA was prepared for RNA-seq (three biological replicates for each group). RNA-seq experiments were performed using Novogene (Beijing, China). Briefly, total RNA was isolated from fresh thoracic aortas tissue using TRIzol. mRNA was then purified from total RNA using poly-T oligo-attached magnetic beads. According to the manufacturer’s recommendations, the sequencing library was generated using NEBNext^®^ UltraTM RNA Library Prep Kit for Illumina^®^ (NEB, lpswich, MA, USA) and the index code was added to the attribute sequence of each sample.

#### 2.9.2. Clustering and Sequencing

After the library was qualified, different libraries were pooled according to the effective concentration and target data volume of the machine, and were then sequenced by Illumina NovaSeq 6000. The end reading of the 150 bp pairing was generated. The basic principle of sequencing was that the synthesis and sequencing were carried out at the same time.

#### 2.9.3. Data Analysis

Through CASAVA base recognition, the image data measured by the high-throughput sequencer were converted into sequence data (reads) by CASAVA base recognition. Raw data (raw reads) in fastq format were first processed by in-house Perl scripts. FeatureCounts (v1.5.0-p3) was used to count the reads numbers mapped to each gene. The differential expression analysis of two conditions/groups (two biological replicates per condition) was performed using the DESeq2 R package (1.20.0). The Gene Ontology (GO) enrichment analysis of differentially expressed genes was implemented by the clusterProfiler R package (3.8.1). We used the clusterProfiler R package (3.8.1) to test the statistical enrichment of differential expression genes in Kyoto Encyclopedia of Genes and Genomes (KEGG) pathways. The above method and process are improved on the basis of the research of Akbar et al. [29].

### 2.10. Determination of ACE Inhibitory Activity In Vitro

The formulation of 11% (*w*/*v*) skimmed milk was sterilized at 105 °C for 15 min. The SR37-3 and SR61-2 bacterial solutions were inoculated into skimmed milk and cultured at 37 °C. The prepared fermented milk with a temperature of 4 °C was centrifuged at 7000× *g* for 10 min. Then, we took the supernatant and adjusted the pH value to 7.5 with 5 mol/L NaOH solution. Next, it was centrifuged at 4 °C, and 11,000× *g* for 3 min, and filtered using 0.45 μM filter membrane for standby. Then, we prepared 50 mmol/L Tris-HCl buffer (containing 0.3 mol/L NaCl), 0.25 U/mL ACE (sigma, St. Louis, MO, USA) and 0.88 mmol/L FAPGG solution (Sigma, St. Louis, MO, USA). Finally, the specific sampling conditions are shown in Table 1. The assay was performed in 96-well microtiter plates.

After the sample was added, it was shaken with the microplate reader (BioTek, Winooski, VT, USA) for 30 s, and we immediately measured the initial absorbance of each sample hole at 340 nm at 37 °C. The initial absorbance was set to a1, b1, c1 and d1, next, the sample was incubated at 37 °C for 15 min, measured again under the same conditions, and the absorbances were set as a2, b2, c2 and d2, respectively. The absorbance reduction values of each sample hole were A = a1 − a2, B = b1 − b2, C = c1 − c2 and D = d1 − d2, respectively. The ACE inhibition rate of PFM was calculated according to the Formula (1) [30].

ACE inhibition rate (%) = [(C − D) − (A − B)]/(C − D) × 100%(1)

### 2.11. Statistical Analysis

The values were presented as the mean ± standard deviation (SD). Data were analyzed by SigmaPlot software (14.0 version). The statistical analysis (Student’s *t*-test or one-way ANOVA tests) was performed and *p* < 0.05 was considered to be statistically significant.

## 3. Results

### 3.1. Changes in BP

**Figure 1:**
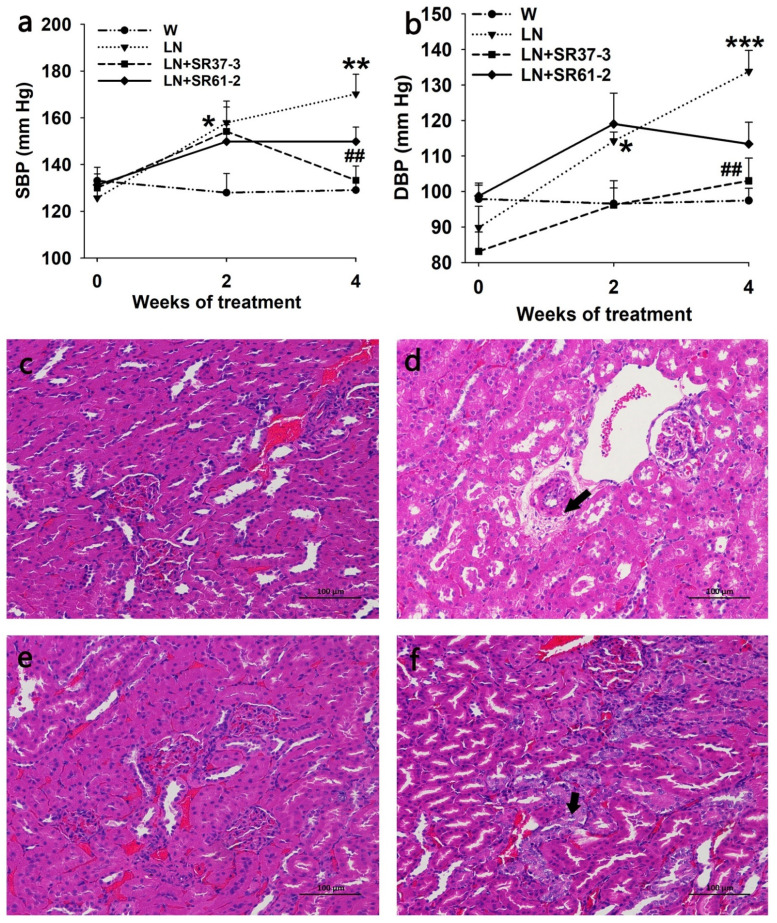
Effects of milk fermented by the *Lactiplantibacillus plantarum* strain SR37-3 (PFM-SR37-3) and milk fermented by the *Lactiplantibacillus plantarum* strain SR61-2 (PFM-SR61-2) on blood pressure and the kidneys in hypertensive rats. The effects of long-term PFM administration on SBP (**a**) and DBP (**b**). *, ** and *** indicate *p* < 0.05, *p* < 0.01 and *p* < 0.001*,* respectively, compared with the W control group, ## indicates *p* < 0.01, compared with the LN control group. The lesion is indicated by the arrow. cont W: animals not treated with N^G^-nitro-L-arginine methyl ester (L-NAME) receiving standard chow; cont LN: L-NAME treated rats receiving standard chow; LN + SR37-3: L-NAME treated rats receiving PFM-SR37-3; LN + SR61-2: L-NAME treated rats receiving PFM-SR61-2. The HE staining of the kidneys; (**c**) W group (control group), (**d**) LN group (model group), (**e**) LN + SR37-3 group and (**f**) LN + SR61-2 group. Values are expressed as mean ± SE (*n* = six rats).

The long-term administration of PFM inhibited a progressive elevation in SBP (170.22 ± 8.40 mmHg and 133.28 ± 6.09 mmHg by LN and LN + SR37-3, respectively, at the end of the 4 weeks; *p* < 0.01 for LN + SR37-3 versus untreated LN) (Figure 1a) and DBP (133.83 ± 5.91 mmHg and 103.00 ± 6.41 mmHg by LN and LN + SR37-3, respectively, at the end of the 4 weeks; *p* < 0.01 for LN + SR37-3 versus untreated LN) (Figure 1b). The consumption of L-NAME in the drinking water for two weeks significantly increased the BP (SBP/DBP) from 125.67 ± 4.26/89.83 ± 6.01 mmHg to 157.89 ± 6.69/114.22 ± 2.55 mmHg (Figure 1a,b). The induced SBP and DBP were also reduced by the *Lactiplantibacillus plantarum* strain SR61-2 (group LN + SR61-2) within the 4-week period; however, they were not reduced at levels with statistical significance (*p* = 0.1 for SBP and *p* = 0.059 for DBP).

### 3.2. PFM Rescues the Morphological Changes in the Kidneys

To study whether PFM could improve renal injury caused by hypertension, we analyzed the morphology in the kidneys based on HE staining. In the W group, the glomeruli were evenly distributed in the cortex, the number of cells in the glomeruli and the matrix were uniform and renal tubular epithelial cells were round and full. No obvious abnormality was observed in the medulla. The connective tissue between the urinary tubules was the renal interstitium without obvious hyperplasia. There was no obvious inflammatory cell infiltration (Figure 1c). In the model group (LN), there was less vacuolar degeneration of renal arteriolar smooth muscle cells than in the control group(W), and microscopic vacuoles were seen in the cytoplasm (black arrow) (Figure 1d). However, no obvious renal abnormalities were observed in the LN + SR37-3 group, and a small amount of renal tubular nucleus enlargement and vacuolation were observed in the LN+ SR61-2 group (black arrow), and no other obvious abnormalities were observed (Figure 1e,f). These results indicated that long-term hypertension could cause chronic kidney damage in rats, and PFM intervention could alleviate the effects to some extent.

### 3.3. Effects of PFM on the Serum Parameters in Hypertensive Rats

**Figure 2:**
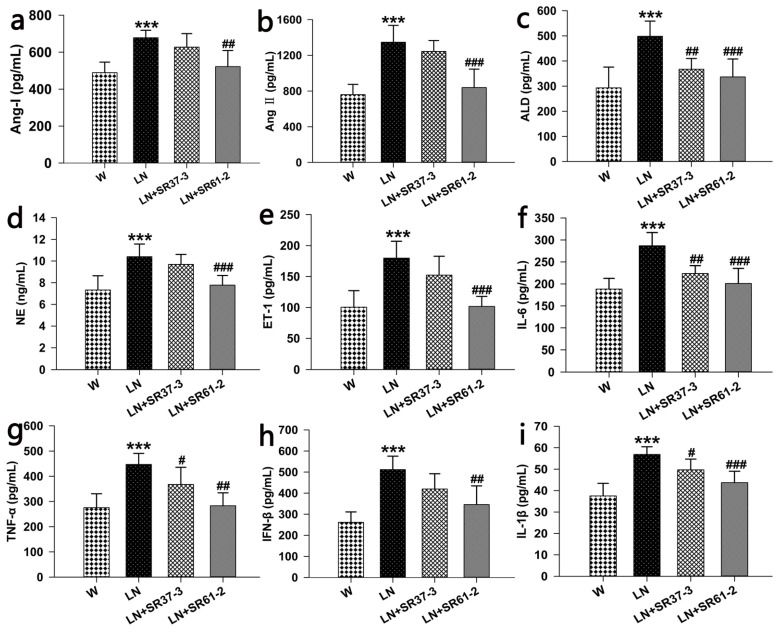
Effects of long-term PFM administration on serum hormones, inflammatory factors and enzymes associated with hypertension. (**a**) for angiotensin I (Ang I), (**b**) for angiotensin II (Ang II), for (**c**) for aldosterone (ALD), (**d**) for norepinephrine (NE), (**e**) for endothelin-1(ET-1), (**f**) for interleukin-6 (IL-6), (**g**) for tumor necrosis factor-α (TNF-α), (**h**) for interferon-β (IFN-β) and (**i**) for interleukin-1β (IL-1β). Values are expressed as mean ± SD (*n* = six rats). *** indicates *p* < 0.001, compared with the W control group; #, ## and ### indicate *p* < 0.05, *p* < 0.01 and *p* < 0.001, respectively, compared with the L-NAME control group. cont W: animals not treated with L-NAME receiving standard chow; cont LN: L-NAME treated rats receiving standard chow; LN + SR37-3: L-NAME treated rats receiving PFM-SR37-3; and LN + SR61-2: L-NAME treated rats receiving PFM-SR61-2.

It was observed that the levels of nine indexes in the LN group treated with L-NAME increased compared with animals not treated with L-NAME (*p* < 0.001, Figure 2a–i). The long-term administration of PFM caused a reduction in angiotensin I (Ang I) (677.99 ± 40.45 pg/mL, 627.09 ± 73.26 pg/mL and 521.78 ± 87.35 pg/mL by LN, LN + SR37-3 and LN + SR61-2, respectively, at the end of the 4 weeks; *p* < 0.01 for LN + SR61-2 versus untreated LN) (Figure 2a), angiotensin II (Ang II) (1346.52 ± 189.8 pg/mL, 1243.52 ± 122.37 pg/mL and 838.36 ± 207.21 pg/mL by LN, LN + SR37-3 and LN + SR37-3, respectively, at the end of the 4 weeks; *p* < 0.001 for LN + SR61-2 versus untreated LN) (Figure 2b), aldosterone (ALD) (498.12 ± 60.69 pg/mL, 367.16 ± 42.98 pg/mL and 336.62 ± 71.26 pg/mL by LN, LN + SR37-3 and LN + SR61-2, respectively, at the end of the 4 weeks; *p* < 0.01 for LN + SR37-3 and *p* < 0.001 LN + SR61-2 versus untreated LN) (Figure 2c), norepinephrine (NE) (10.4 ± 1.17 ng/mL, 9.69 ± 0.92 ng/mL and 7.78 ± 0.89 ng/mL by LN, LN + SR37-3 and LN + SR61-2, respectively, at the end of the 4 weeks, *p* < 0.001 for LN + SR61-2 versus untreated LN) (Figure 2d), endothelin-1(ET-1) (179.68 ± 27.21 pg/mL, 152.24 ± 30.47 pg/mL and 101.62 ± 16.24 pg/mL by LN, LN + SR37-3 and LN + SR61-2, respectively, at the end of the 4 weeks; *p* < 0.001 for LN + SR61-2 versus untreated LN) (Figure 2e), interleukin-6(IL-6) (286.87 ± 29.95 pg/mL, 223.57 ± 17.99 pg/mL and 200.91 ± 34.4 pg/mL by LN, LN + SR37-3 and LN + SR61-2, respectively, at the end of the 4 weeks; *p* < 0.01 for LN + SR37-3 and *p* < 0.001 for LN + SR61-2 versus untreated LN) (Figure 2f), tumor necrosis factor-α (TNF-α) (446.85 ± 44.08 pg/mL, 367.47 ± 68.09 pg/mL and 283.23 ± 51.14 pg/mL by LN, LN + SR37-3 and LN + SR61-2, respectively, at the end of the 4 weeks; *p* < 0.05 for LN + SR37-3 and *p* < 0.001 for LN + SR61-2 versus untreated LN) (Figure 2g), interferon-β (IFN-β) (511.61 ± 63.81 pg/mL, 419.71 ± 72.52 pg/mL and 345.92 ± 88.56 pg/mL by LN, LN + SR37-3 and LN + SR61-2, respectively, at the end of the 4 weeks; *p* < 0.01 for LN + SR61-2 versus untreated LN) (Figure 2h), interleukin-1β (IL-1β) (56.87 ± 3.58 pg/mL, 49.68 ± 4.99 pg/mL and 43.69 ± 5.35 pg/mL by LN, LN + SR37-3 and LN + SR61-2, respectively, at the end of the 4 weeks; *p* < 0.05 for LN + SR37-3 and *p* < 0.001 for LN + SR61-2 versus untreated LN) (Figure 2i). Therefore, we speculate that the decrease in BP in rats may be related to inflammation and achieved by inhibiting the ACE activity of the renin–angiotensin system [31].

### 3.4. PFM Alleviates Intestinal Microbiota Dysbiosis in Hypertensive Rats

**Figure 3:**
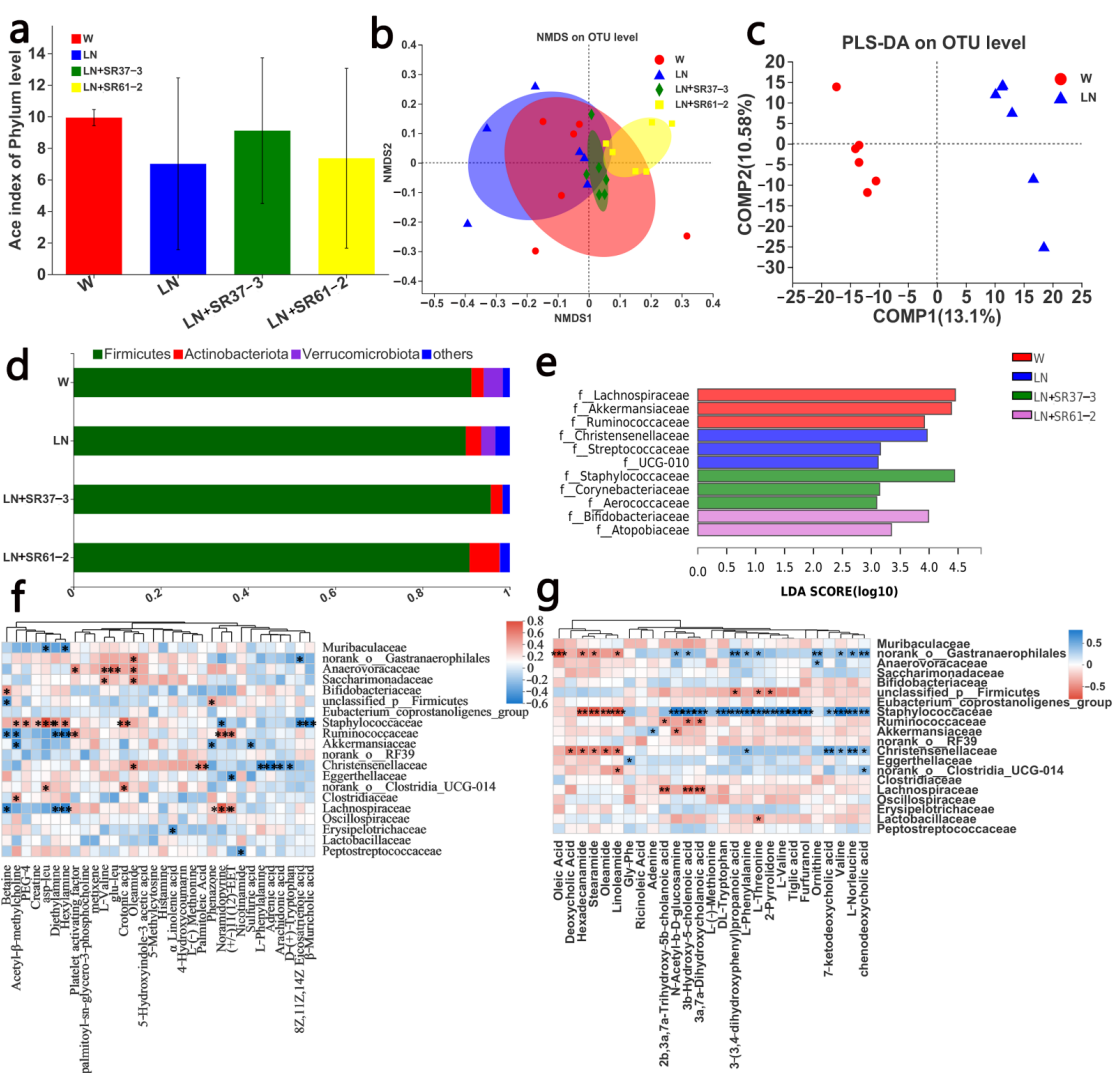
PFM regulated the bacterial community of the cecum microbiota. (**a**) ACE Index; (**b**) NMDS; (**c**) PLS-DA on the OTU level between W and LN; (**d**) relative abundance richness at phyla level; and (**e**) LEFSE on family level. (**f**) Correlation heatmap between intestinal microorganisms and environmental factors (serum differential metabolites), * *p* < 0.05, **p < 0.01, *** *p* < 0.001. (**g**) Correlation heatmap between intestinal microorganisms and environmental factors (differential metabolites of cecal contents), * *p* < 0.05, ** *p* < 0.01, *** *p* < 0.001. cont W: animals not treated with L-NAME receiving standard chow; cont LN: L-NAME treated rats receiving standard chow; LN + SR37-3: L-NAME treated rats receiving PFM-SR37-3; LN + SR61-2: L-NAME treated rats receiving PFM-SR61-2.

The pathogenesis of hypertension is complex and diverse, and may include intestinal microbial changes, sympathetic nervous system excitation and inflammation. Therefore, in the following series of experiments, we studied the changes in the intestinal microbial composition, the differences in blood and fecal metabolites and the gene expression of the thoracic aorta to explore the possible mechanism of BP changes caused by the changes in the intestinal flora. Since intestinal dysbiosis is related to hypertension, we investigated the regulatory effects of PFM on the composition of gut microbiota by a *16S rDNA* sequencing assay of fecal bacteria. The ACE Index was used to reflect the alpha diversity of the intestinal microbiota. Compared with the W group, the LN group decreased the ACE Index (*p* = 0.2187), while PFM-SR37-3 and PFM-SR61-2 intervention improved the ACE Index and the richness of the intestinal microbiota (*p* = 0.5844 and *p* = 0.915, respectively), and there was not a significantly difference. Previously reported results also showed that the Shannon index, the Simpson index and Pielou evenness were not significantly different between the control group and the hypertension group [32]. Nonmetric multidimensional scaling (NMDS) analysis also showed that the four groups were clustered in different places (Figure 3b), with the most abundant phyla of the four groups being Firmicutes (Figure 3d). The rats in the LN group were suffered from hypertension, which exhibited a disordered and scattered distribution of intestinal microbiota. Compared with the LN group, the distribution of intestinal microbiota in the LN + SR37-3 group was relatively stable and concentrated, and was close to the control group. The difference in the microbiota between the LN and LN + SR37-3 group indicated that PFM-SR37-3 had a positive effect on the intestinal microbiota. PLS-DA analysis also showed that there was a significant separation in the OTU level between the disease group and the control group (Figure 3c). Linear discriminant analysis effect size (LEfSe) analysis was adopted to explore the abundance of the dominant microbe in each group (Figure 3e). In comparing of the W group with the LN group at the family level, we found that the relative abundances of *Lachnospiraceae*, *Akkermansiaceae* and *Ruminococcaceae* were statistically higher in the W group, while *Christensenellaceae*, *Streptococcaceae* and UUG-010 were the dominant microbes in the LN group. It is pertinent to point out that the abundance of *Akkermansia* in healthy rats was high, which is negatively correlated with hypertension [32]. Studies showed that a decrease in *Akkermansia* is related to obesity and diabetes, and the introduction of Akkermansia could reverse the metabolic disorder and improve inflammation in diabetics and obese patients [33]. However, studies have also shown that the increase in *Streptococcaceae* is significantly correlated with myocardial infarction [34]. After PFM-SR61-2 intervention, the beneficial bacteria *Bifidobacteriaceae* significantly displayed an increased abundance. *Bifidobacterium* has antibacterial, antiinfection, immune regulation, cholesterol lowering and other physiological functions. Research has shown that the intestinal *Bifidobacterium* is significantly reduced in patients and animals with hypertension [32]. At the same time, the abundance of *Atopobiaceae* also increased, which was positively correlated with the production of volatile fatty acids [35].

### 3.5. Effect of PFM on Metabolic Profiles

**Figure 4:**
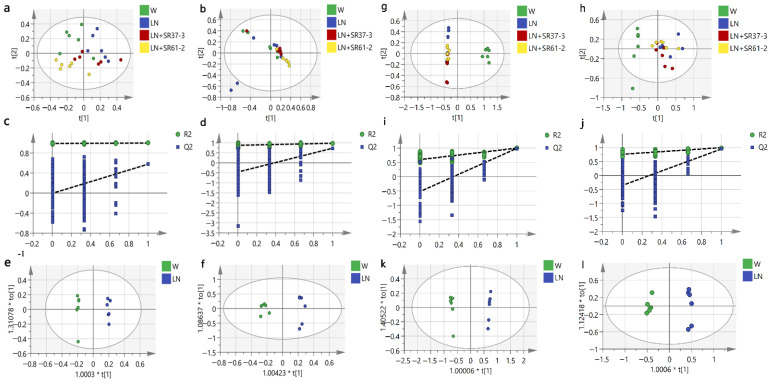
The analysis of the LC-MS of serum samples of hypertensive rats (**a**–**f**). The distribution of the PCA score plots for the W, LN, LN + SR37-3 and SR61-2 groups in the positive model (**a**) and negative model (**b**). Permutation test charts for the OPLS-DA model between W and LN of the metabolomics data set in the positive (R^2^ = (0, 0.989); Q^2^ = (0, −0.00648)) model (**c**) and the negative (R^2^ = (0, 0.876); Q^2^ = (0, −0.458)) model (**d**). The OPLS-DA score plot of the metabolomics data set in the positive (R^2^Y = 0.997; Q^2^Y = 0.575) model (**e**) and negative (R^2^Y = 0.981; Q^2^Y = 0.732) model (**f**). The analysis of the LC-MS of cecal contents samples of hypertensive rats (**g**–**l**). The PCA score plots distribution for the W, LN, LN + SR37-3 and SR61-2 group in the positive model (**g**) and negative model (**h**). Permutation test charts for the OPLS-DA model between the W and LN of the metabolomics data set in the positive (R^2^ = (0, 0.597); Q^2^ = (0, −0.52)) model (**i**) and negative (R^2^ = (0, 0.759); Q^2^ = (0, −0.355)) model (**j**). The OPLS-DA score plot of the metabolomics data set in the positive (R^2^Y = 0.997; Q^2^Y = 0.99) model (**k**) and negative (R^2^Y = 0.994; Q^2^Y = 0.945) model (**l**). cont W: animals not treated with L-NAME receiving standard chow; cont LN: L-NAME-treated rats receiving standard chow; LN + SR37-3: L-NAME-treated rats receiving PFM-SR37-3; and LN + SR61-2: L-NAME treated rats receiving PFM-SR61-2. t[1] is the weight of the regression coefficient for the predicted principal component (abscissa). The data next to “*” represents the weight value. “*” Not practical.

**Figure 5:**
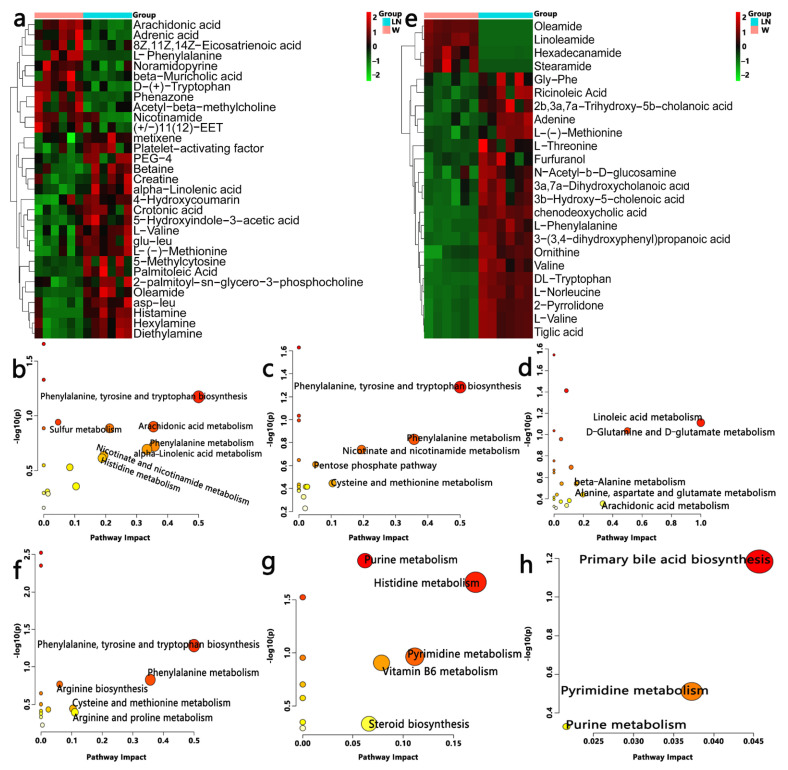
Effects of PFM-SR37-3 and PFM-SR61-2 on gut and blood metabolomes. (**a**,**e**) are heatmaps between W and LN groups constructed from the important metabolites of serum and cecal samples, respectively. A hierarchically clustered heat map showing the relative increase/decrease in metabolite contents between individual samples and their similarity. Columns correspond to different groups, and rows correspond to the altered metabolites. Color keys indicates the metabolite expression value; green is the lowest and red is the highest. (**b**–**d**) are metabolomic views of serum samples from the pathway analysis performed using MetaboAnalyst between the W and LN groups, between the LN and LN + SR37-3 groups, and between the LN and LN + SR61-2 groups, respectively. On the other hand, (**f**–**h**) are metabolomic views of cecal contents samples. cont W: animals not treated with L-NAME receiving standard chow; cont LN: L-NAME-treated rats receiving standard chow; LN + SR37-3: L-NAME-treated rats receiving PFM-SR37-3; LN + SR61-2: L-NAME-treated rats receiving PFM-SR61-2.

To understand the antihypertensive effect of PFM from the perspective of host metabolism, we used nontargeted metabonomics to analyze the serum and cecal metabolome of PFM-treated hypertensive rats. From the PCA plot scores, we could observe that W, LN, LN + SR37-3 and LN + 61-2 groups were in different locations in the positive model (Figure 4a,g); however, there was no significant separation between the groups in the negative ion mode (Figure 4b,h). Whether it is serum or cecal content, the OPLS-DA score plots between W and LN groups in positive and negative ion modes were shown separately with Figure 4e,f,k,l. From the distribution trend we can further confirm that the hypertension model was set up successfully with L-NAME. The chemical identification of these feature metabolites was achieved by a local database as well as online databases including HMDB (www.hmdb.ca, accessed on 17 October 2021) [36] according to mass fragmentation patterns and isotope peak ratios. To further confirm significant variables, the first principal component of variable importance projection (VIP) was calculated, and a VIP value of > 1 and *p* < 0.05 on Student’s *t*-test were considered to indicate a statistical difference. The results of serum samples showed that 31 metabolites between W and LN, 21 metabolites between LN and LN + SR37-3 and 28 metabolites between LN and LN + SR61-2 were identified, which are shown in Supplement Table S1, including amino acids, fatty acids, amide derivatives and choline derivatives. The results of cecal samples showed that 27 metabolites between W and LN, 31 metabolites between LN and LN + SR37-3 and 24 metabolites between LN and LN + SR61-2 were identified, which are shown in Supplement Table S2, including amino acids, fatty acids, amide derivatives, bile derivatives, pyrimidines, choline derivatives, etc. To provide a more comprehensive insights into the mechanisms of hypertension, we performed a pathway analysis of feature metabolites using MetaboAnalyst 4.0 (www.metaboanalyst.ca accessed on 15 January 2022) [37]. In the serum sample, on account of 30 significantly changed metabolites between W and LN, seven metabolic pathways that were significantly influenced by hypertension are founded (Figure 5b) and three of them are amino acids metabolism. Moreover, this is similar to the results in the sample of cecal contents, in which the first five metabolic pathways are the amino acid metabolism (Figure 5f). Based on 22 significantly changed metabolites between LN and LN + SR37-3, five metabolic pathways that were significantly influenced by PFM-SR37-3 are founded (Figure 5c), three of which belong to the amino acid metabolism. However, the histidine metabolism and pyrimidine metabolism are mainly involved in fecal samples (Figure 5g). In view of 29 significantly changed metabolites between LN and LN + SR61-2, five metabolic pathways that were significantly influenced by PFM-SR61-2 are founded (Figure 5d) and they mainly involve fatty acid and amino acid metabolisms; however, in fecal samples, it is mainly related to the biosynthesis of primary bile acids (Figure 5h). A Heatmap visualization of potentially important candidates showed that there was a distinct separation between the LN group and W group in serum (Figure 5a) and cecal contents (Figure 5e) samples. However, most serum and cecal content metabolites had the same trend in the control group and the model group. The metabolites were separated into two big clusters (up and down). The metabolites in the “up” cluster were mainly enriched in the control group, while the metabolites in the “down” cluster were mainly enriched in the disease group. Specifically, we found that L-phenylalanine, L-methionine and L-valine are three different metabolites involved in intestinal and blood metabolism at the same time. Phenylalanine, tyrosine and tryptophan biosynthesis and phenylalanine metabolism are common metabolic pathways of serum and cecal contents, which may be related to the development mechanism of the disease. L-phenylalanine could improve vascular tetrahydrobiopterin However, compared with the W group, the LN group had more L-phenylalanine in the faces. Studies have shown that bacteria in the large intestine metabolize unabsorbed phenylalanine into phenylpyruvate to produce polyacrylic acid (PAA). PAA is then circulated through the portal venous system to be metabolized in the liver to produce PAGln [38].

For the further investigation of the correlation between the gut microbiota and other sample indicators, the analysis of the environmental factors was adopted to establish a redundancy analysis (RDA) model for microbiota samples and the main factors from the metabolome analysis and we drew the correlation heat map. Here, there was a good correlation between metabolomes and gut microbiota in blood or cecal contents (Figure 3f,g). Most of the metabolites were positively correlated with intestinal flora. L-valine in the serum metabolome was positively correlated with anaerovoracaceae and saccharimonadaceae, while L-valine was positively correlated with staphylococcaceae in the cecal content metabolome. L-phenylalanine in the serum metabolome was positively correlated with Christensenellaceae, but in the cecal content metabolome, L-phenylalanine was positively correlated with *Norank_Gastranaerophilales, Christensenellaceae and Staphylococcaceae*. The data suggested that the cecal contents metabolome, the blood metabolome and gut microbiota interacted together, which further indicated that the occurrence of hypertension is related to intestinal flora disorder or flora metabolism (Figure 3f,g).

### 3.6. Transcriptome Profiles Reveals the Effect of PFM on the Thoracic Aorta of Hypertensive Rats

**Figure 6:**
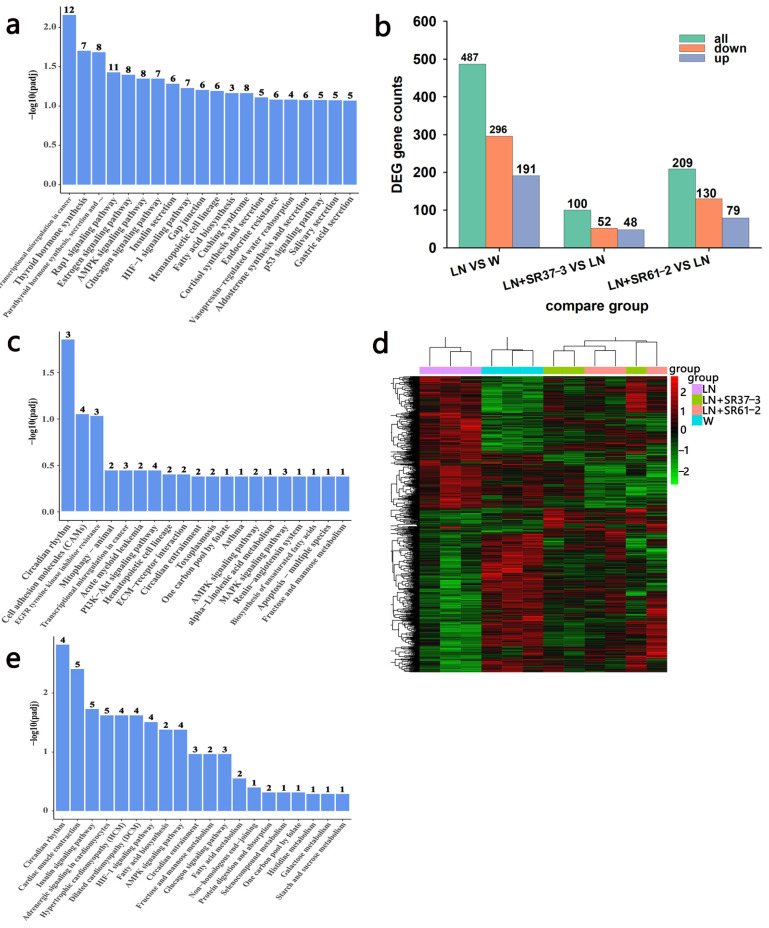
The functional enrichment and expression profile of DEGs in the thoracic aorta. (**a**) The KEGG PATHWAY functional enrichment of down-regulated DEGs between the disease group and the control group. (**b**) The bar chart shows all significant DEGs in each comparison group, including up-regulated or down-regulated genes. (**c**) The KEGG PATHWAY functional enrichment of up-regulated DEGs between the LN + SR31-3 group and the LN group. (**d**) Heatmap showing the differentially expressed lncRNAs between different groups. (**e**) KEGG PATHWAY functional enrichment of up-regulated DEGs between the LN + SR61-2 group and the LN group. cont W: animals not treated with L-NAME receiving standard chow; cont LN: L-NAME-treated rats receiving standard chow; LN + SR37-3: L-NAME treated rats receiving PFM-SR37-3; LN + SR61-2: L-NAME-treated rats receiving PFM-SR61-2.

**Figure 7:**
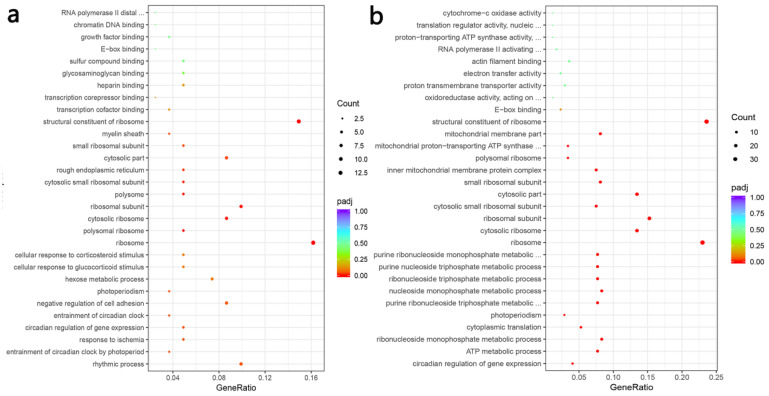
Gene ontology functional enrichment. Gene ontology functional enrichment of all DEGs in LN + SR37-3 vs. LN (**a**) and LN + SR61-2 vs. LN (**b**). cont LN: L-NAME treated rats receiving standard chow; LN + SR37-3: L-NAME treated rats receiving PFM-SR37-3; LN + SR61-2: L-NAME treated rats receiving PFM-SR61-2.

A blood vessel is an important target organ of hypertension and blood vessels plays an important role in the occurrence and development of blood pressure and cardiovascular disease. Investigating the alteration of thoracic aortic transcriptome profiles underlying the hypertension and PFM administration in the hypertension model could provide comprehensive insights for understanding the mechanisms of PFM for improving cardiovascular health. The transcriptome analysis demonstrated that the expression levels of 487 genes were significantly changed in the group comparison of hypertension vs. non-hypertension, and 61% of which were down-regulated (Figure 6b). It is worth noting that PFM supplementation regulated the expression of DEGs (Figure 6d). Furthermore, we respectively performed DEG’s Gene Ontology (GO) term enrichment and KEGG pathway enrichment on the LN vs. control group, the LN + SR37-3 vs. LN group and the LN + SR61-2 vs. LN group to reveal the overall functional enrichment characteristics of all DEGs. As shown in Figure 6a, the KEGG enrichment pathway analysis of DEGs downregulating in the LN group was mainly involved in the synthesis and secretion of hormones. Most DEGs were related to substances related to metabolic diseases, such as thyroid hormone synthesis, the AMPK signaling pathway, the glucagon signaling pathway, insulin secretion, fatty acid biosynthesis, cortisol synthesis and secretion and vasopressin-regulated water reabsorption (Figure 6a). However, some of these hormone-synthesis-related genes had higher expression in PFM-SR37-3 and PFM-SR61-2-treatment rats (Figure 6c,e). The KEGG enrichment pathway analysis of DEGs upregulating in the LN + SR37-3 group showed that PFM-SR37-3 supplementation improved the AMPK signaling pathway, alpha-linolenic acid metabolism, the MAPK signaling pathway, renin-angiotensin system and the biosynthesis of unsaturated fatty acids induced by long-term LN feeding (Figure 6c). Meanwhile, the KEGG enrichment pathway analysis of DEGs upregulating in the LN + SR61-2 group showed that PFM-SR61-2 supplementation improved the insulin signaling pathway, fatty acid biosynthesis, the AMPK signaling pathway, the glucagon signaling pathway, fatty acid metabolism and histidine metabolism (Figure 6e). In particular, it must be noted particularly that both PFM-SR37-3 and PFM-SR61-2 intervention upregulated the DEGs associated with AMPK and circadian rhythms. Circadian rhythms are the most prevalent DEGs, which are supported by their role in the blood pressure. Moreover, recent studies have shown that circadian clock genes are involved in the regulation of the heart, kidneys, vasculature and metabolic organs, which are all critical to the regulation of BP [39]. We did not perform KEGG analysis on the upregulated DEGs in the LN group and the downregulated DEGs in the LN + SR37-3 and LN + SR61-2 groups because the GO term of these DEGs did not involve metabolic disease or cardiovascular disease. Compared with the hypertension group, PFM-SR37-3 treatment significantly altered the expression levels of 100 genes including 52 down-regulated and 48 up-regulated genes, which were significantly enriched in ribosome synthesis (structural constituent/subunit), the cellular response to glucocorticoid stimulus and the cellular response to corticosteroid stimulus by GO analysis (Figure 7a). Meanwhile, PFM-SR61-2 treatment significantly altered the expression levels of 209 genes including 130 down-regulated and 79 up-regulated genes, which were significantly enriched in ribosome synthesis (structural constituent/subunit) and the ribonucleoside metabolic process (ribonucleoside monophosphate, purine ribonucleoside triphosphate, nucleoside monophosphate, ribonucleoside triphosphate, purine nucleoside triphosphate, purine ribonucleoside monophosphate) by GO analysis (Figure 7b).

### 3.7. ACE Inhibition Rate of PFM Supernatant In Vitro

The ACE inhibition rate was used as an indicator to measure the potential antihypertensive function of two strains of *Lactiplantibacillus plantarum* in vitro. The results showed that the ACE inhibition rates of *Lactiplantibacillus plantarum* SR37-3 and *Lactiplantibacillus plantarum* SR61-2 were 70.5% and 68.9% respectively.

## 4. Discussion

This study indicated that PFM-SR37-3 and PFM-SR61-2 have antihypertensive potential in vivo and in vitro, and they could alleviate renal injury caused by hypertension. Using the L-NAME model, we showed that PFM-SR37-3 and PFM-SR61-2 significantly reduced RAS system-related substances in serum and improved blood pressure. Additionally, PFM intervention changed the gut microbiome; in particular, it increased the abundance of probiotics such as *Bifidobacteriaceae* and *Atopobiaceae*. Furthermore, we found that PFM intervention regulated L-phenylalanine, L-methionine and L-valine metabolites in intestinal and blood circulation and up-regulated the expression of fatty acid biosynthesis, the AMPK signaling pathway and circadian rhythms related genes in aortic vessels. These findings by the jointing analysis of multiomics demonstrated for the first time that PFM could effectively attenuate hypertension and protect cardiovascular health. Our results provided new insight into the antihypertensive properties of PFM, showing gut microbiota-metabolites-mediated mechanisms of action for this probiotic.

Environmental factors are the determinants that directly influence the occurrence and development of hypertension. How to reduce the influence of environmental factors to effectively slow down the development of hypertension is the primary challenge in the management of the disease, and the most studied environmental factors are dietary factors [40]. Among functional foods, those containing probiotics lead in the market, but dairy products are considered optimum carriers for probiotics. In the past few years, fermented milks, among the fermented foods, have been widely promoted in the media due to their promising health benefits [41]. Gut microbiota are closely related to human health. Accumulated studies revealed that the occurrence of hypertension might be associated with gut microbial dysbiosis [42,43]. Bhanu et al. [44] found that gut microbial dysbiosis played a causal role in the development of hypertension in rat models of obstructive sleep apnea (OSA). A functional analysis of the dysregulated gut microbiota of OSA rats showed that SCFA-producing bacteria were absent. However, Gomez-Guzman et al. showed that SBP decreased significantly and renal hypertrophy was improved in SHR by probiotic intervention [45]. Previous studies showed the antihypertensive effect of fermented milks, and the long-term consumption of biologically active fermented dairy products might have a protective effect on the development of cardiovascular disease, including hypertension [46]. However, the underlying mechanisms of such effects have not been completely elucidated. Based on the reported evidence of gut dysbiosis in hypertension, combined with the known intestinal eubiotic and antihypertensive properties of PFM, we hypothesized that PFM can partially inactivate the RAS system and attenuate inflammation while also reducing blood pressure in hypertensive animals by improving the gut microenvironment. Hypertension can lead to many complications, including hypertensive nephropathy. In addition, hypertension can result in damage within the kidneys, eventually leading to end-stagerenal disease. Previous studies demonstrated the antihypertensive effect of fermented milks [47,48]. Moreover, a study also reported that blueberries fermented by *Lactiplantibacillus plantarum* had antihypertensive activity in hypertensive rats induced by L-NAME [18]. Consistent with these works, we found that fermented milk with *Lactiplantibacillus plantarum* SR37-3 and SR61-2 significantly prevented the rise of BP and attenuated the renal pathological damage of the L-NAME rats. This suggested that PFM could alleviate hypertension and its complications, as well as renal damage. Modeling rats with L-NAME consistently raised blood pressure, whereas our probiotic fermented milk treatment suppressed the BP rise. However, due to the specificity of the strain, the potential antihypertensive effect of LN + SR37-3 strain is different from LN + SR61-2, and the long-term treatment effect of PFM-SR37-3 is better than that of PFM-SR61-2. LN + SR37-3 treatment increased then decreased after 4 weeks, while other treatments remained increased (Figure 1a). LN + SR61-2 treatment increased then decreased after 4 weeks, while other treatments remained increased (Figure 1b). However, despite this, the DBP of the PFM-SR37-3 treatment group was still lower than that of the PFM-SR61-2 treatment group. The study by Gomez-Guzman et al. also showed that when different bacteria were used, the effect on hypertension was different [45]. Alhaj’s research also showed that the amount of ACE-inhibiting peptides produced by different strains is not consistent [8]. Probiotics release ACE-inhibiting peptides through their unique proteolytic system, which consists of cellular envelope proteases (CEPs), transport systems and intracellular peptidases. Currently, the reported ACE inhibitory effects of food-derived peptides are divided into three modes: competitive, uncompetitive and non-competitive. Different probiotic strains have different proteolytic activity and proteolytic systems [49]. Therefore, ACE inhibitory peptides with different activities will be produced by different strains, resulting in different antihypertensive effects. In addition, because of the heterogeneity between different strains, their metabolites and their effects on the gut microbiota are different, so their antihypertensive potentials are different.

To clarify the role of the renin–angiotensin system (RAS) in this effect of PFM, the expressions of Ang I, Ang II and ALD in the serum were determined. Our results showed that PFM might partially inactivate RAS, resulting in reduced angiotensin II production and effects. The expression of ET-1 and NE in the serum was also determined. ET-1, thought to be a potent vasoconstrictor, was overexpressed in the vasculature of different hypertensive models. In addition to lowering BP, ET receptor antagonists could also reduce vascular growth. NE is related to the excitation of the sympathetic nerve, which can increase BP and the heart rate. After PFM intervention, the contents of ET-1 and NE in the serum of hypertensive rats decreased significantly compared with the model group. We speculate that PFM can reduce vascular endothelial dysfunction and stress response. Santisteban et al. [43] proposed the influence of the brain–gut–bone marrow interaction on hypertension. The interaction between the three caused a persistent inflammatory response resulting in BP elevation. Therefore, alleviating the inflammatory response may reduce BP. Previous studies reported that the level of inflammatory factors was related to the BP level and target organ injury of hypertension [50,51]. Consistent with previous studies, the level of inflammatory factors in the hypertension group was significantly higher than that in the control group, but decreased significantly after PFM-SR37-3 and PFM-SR61-2 intervention. A body of evidence showed that PFM had ACE inhibition activity. We measured the ACE inhibition rate of its supernatant to evaluate the antihypertensive activity of PFM in vitro. Consistent with the previous research results [52,53], both PFM-37-3 and PFM-61-2 have high ACE inhibitory activity. It was shown that PFM had favorable antihypertensive potential.

One possible mechanism by which probiotics lower blood pressure is to modulate the production of gut metabolites by modulating gut microbiota. Intestinal microbial metabolism may play an important role in cardiovascular diseases. For example, previous studies showed that trimethylamine N-oxide (TMAO) could significantly affect the metabolic level of tryptophan in feces, thus promoting the development of atherosclerosis. SR37-3 and SR61-2 interventions increased the abundance of probiotics such as *Bifidobacteriaceae* and *Atopobiaceae*, modulated gut microbiota and altered metabolite levels. It has always been a matter of concern that intestinal microbial metabolites can cross the intestinal barrier and reach the blood. By combining fecal and blood non-targeted metabolomics, we found that L-phenylalanine, L-methionine and L-valine were three different metabolites involved in the intestines and blood metabolism at the same time, suggesting that intestinal microbiota might affect the amino acid metabolism and lead to hypertension. A controlled study of the association between metabolomes and hypertension risk showed that phenylalanine, tyrosine and tryptophan biosynthesis were the most relevant metabolic pathways [54]. L-phenylalanine could improve vascular tetrahydrobiopterin, restores NO, reduce superoxide and enhances vascular function in spontaneously hypertensive rats by activating the protein complex (GCH1-GFRP) involved in the biosynthesis of tetrahydrobiopterin [55]. Additionally, L-phenylalanine could attenuate high salt-induced hypertension in Dahl SS rats [56]. Studies have found that PAGln can enhance the stimulatory response of platelets to various agonists and intracellular calcium release [57]. In addition, PAGln can enhance platelet reactivity and thrombosis through G protein-coupled receptors, leading to cardiovascular disease [58]. Robin et al. reported that a methionine-enriched diet induced a hyperhomocysteinaemia and an elevated SBP in Wistar and Sprague Dawley rats [59,60]. Methionine can significantly increase the concentration of ACE in blood and cause hypertension [61]. Findings indicated that a higher intake of branched chain amino acids intake, especially valine, is associated with a higher risk of incident hypertension [62]. Moreover, a relevant study showed that tyrosine was an identified biomarker for essential hypertension. More tyrosine and methionine were required for sympathetic activation, which might be an important mechanism of essential hypertension [63]. However, we did not explore the role of intestinal organs here, because intestinal epithelial cells were only involved in the absorption process and did not affect the biochemical response of intestinal cells here. Gut-derived microbial metabolites can cross the intestinal barrier and enter the blood circulation to exert biological effects [64]. These metabolites were produced by bacteria breaking down the contents. We will explore whether microbial small molecule metabolites can interact with host cells to generate signal transduction with distant target organs in subsequent studies.

Next, we applied RNA-sequencing to investigate the underlying mechanisms of PFM on the function of the aorta, which was the one of the target organs affected by hypertension. Notably, we noted a significant increase in the expression of genes involved in circadian rhythms after PFM-SR37-3 and PFM-SR61-2 intervention. Previous studies revealed an essential role of circadian rhythms in regulating BP. As we all know, human BP shows a certain circadian rhythm and circulating RAS has a circadian rhythm [65]. Tubular sodium reabsorption stimulated by the activation of the intrarenal renin angiotensin system (RAS) contributes to the development of the disturbed circadian BP rhythm. The abnormal circadian rhythm of BP can contribute to target organ damage and cardiovascular and cerebrovascular events. In our study, PFM-SR37-3 mainly upregulated the expression of Per1, Per2 and Nr1d1, and PFM-SR61-2 mainly upregulated the expression of Per1, Per2, Per3 and Bhlhe40. A study showed that Per1 controls circadian BP rhythms mediated via regulating the distal nephron Na transporter gene [66]. A previous study reported that Per1, Per2, Per3 and Reverba5(Nr1d1) mRNA tended to be lower in SHR adrenal glands than in controls, which might affect the transcriptional regulation of clock-controlled genes, and steroid hormone secretion by the adrenal gland [67]. A study suggested that TNF- α-induced vascular smooth muscle cell proliferation and oxidative stress could be inhibited by promoting Bhlhe40 expression, thereby ameliorating of intimal hyperplasia [68].

To sum up, our results showed that the long-term use of probiotic yogurt could lower BP and attenuate renal damage by inactivating the RAS system and improving intestinal microbiota, increasing the abundance of probiotics and regulating the metabolic levels of L-phenylalanine, L-methionine and L-valine in the intestine and blood. The results of target organ vascular transcriptome sequencing showed that the up regulation of genes related to circadian rhythm may play an important role in antihypertension. Our findings indicated that PFM had an antihypertensive effect by acting on the RAS system and is mediated by gut microbiota and the antihypertensive mechanism was preliminarily explored, which might provide useful clues for developing new dietary strategies to prevent hypertension.

There is still some controversy about the benefits of probiotics. For example, some animal experiments and human clinical trials showed that probiotics were not beneficial for cardiovascular disease. Part of this discrepancy may be related to probiotic strains, doses, and heterogeneity among participants. In addition, most of the current research on probiotics is focused on the population with specific diseases, and the effect of probiotics on healthy individuals needs further research to be determined. Therefore, in the future, we need to identify specific strains, not just colony communities. We need to elucidate the functions of metabolites produced by microorganisms and their downstream pathways/molecules, and shift from correlation to causality, and from bacterial characteristics to molecular mechanisms. There are not many foods that use probiotics to lower blood pressure in the world. One reason for this is that the antihypertensive mechanism of probiotics is not clear enough, and the other is that they are difficult to use in food. Thus, this direction still needs to combine a large amount of basic research with clinical research to provide more data information. We need to continuously optimize microbiota detection techniques and conduct more explorations on the microbiota’s relationship between probiotics and hypertension to provide more information and to find more effective microbiota or microbiota metabolites to develop foods with blood-pressure-lowering functions. However, some limitations of our study deserve mention. We found a way to intervene in hypertension. In the future, further research is needed to determine whether probiotics pre-fermentation play a role.

## Figures and Tables

**Table 1:** Determination of the ACE inhibition rate.

Reagent	a	b	c	d
ACE	10 μL	-	10 μL	-
Milk serum sample	10 μL	10 μL	-	-
Tris-HCl	-	10 μL	10 μL	20 μL
FAPGG	150 μL	150 μL	150 μL	150 μL

## Data Availability

The authors declare that all of the data and the material used in this study are available within this article. All data generated or analyzed in this study can be obtained from the authors upon reasonable request.

## Supplementary Materials

The following supporting information can be downloaded at: https://www.mdpi.com/article/10.3390/foods11152332/s1, Table S1: Detection of different metabolites in serum samples; Table S2: Detection of different metabolites in cecal contents samples.

## Abbreviations

Abbreviation	Full name
ACE	Angiotensin Converting Enzyme
ALD	aldosterone
Ang Ⅰ	angiotensin I
Ang II	angiotensin II
BP	blood pressure
CVD	cardiovascular disease
DBP	diastolic BP
ELISA	enzyme linked immunosorbent assay
ET-1	endothelin-1
GO	Gene Ontology
HE	Hematoxylin-eosin
HESI	heating electrospray ion source
IFN-β	interferon-β
IL-1β	interleukin-1β
IL-6	interleukin-6
IPP	Ile-Pro-Pro
KEGG	Kyoto Encyclopedia of Genes and Genomes
LEfSe	linear discriminant analysis effect size
L-NAME	N^G^-nitro-L-arginine methyl ester
PFM-SR37-3	milk fermented by *Lactiplantibacillus plantarum* strain SR37-3
PFM-SR61-2	milk fermented by *Lactiplantibacillus plantarum* strain SR61-2
NE	norepinephrine
NMDS	Nonmetric multidimensional scaling
OPLS-DA	orthogonal least squares discriminant analysis
OSA	obstructive sleep apnea
OTUs	Operational taxonomic units
PAA	produce polyacrylic acid
PCA	principal component analysis
PCR	Polymerase Chain Reaction
PFM	Probiotics fermented milk
RAS	renin–angiotensin system
RCTs	randomized controlled trials
RDA	Redundancy Analysis
SBP	systolic BP
SD	standard deviation
SHR	spontaneously hypertensive rats
SPF	specific pathogen-free
TMAO	trimethylamine N-oxide
TNF-α	tumor necrosis factor α
VIP	variable importance projection
VPP	Val-Pro-Pro

## References

[1] Staessen J.A. (2014). Age-specificity of blood-pressure-associated complications. Nat. Rev. Cardiol..

[2] Kahan T. (2014). Focus on blood pressure as a major risk factor. Lancet.

[3] Chen M., Sun Q., Giovannucci E., Mozaffarian D., Manson J.E., Willett W.C., Hu F.B. (2014). Dairy Consumption and Risk of Type 2 Diabetes: 3 Cohorts of Us Adults and an Updated Meta-Analysis. BMC Med..

[4] Hou Q., Li C., Liu Y., Li W., Chen Y., Siqinbateer, Bao Y., Saqila W., Zhang H., Menghe B. (2018). Koumiss consumption modulates gut microbiota, increases plasma high density cholesterol, decreases immunoglobulin G and albumin. J. Funct. Foods.

[5] Li C., Kwok L.-Y., Mi Z., Bala J., Xue J., Yang J., Ma Y., Zhang H., Chen Y. (2017). Characterization of the angiotensin-converting enzyme inhibitory activity of fermented milks produced with *Lactobacillus casei*. J. Dairy Sci..

[6] Tillisch K., Labus J., Kilpatrick L., Jiang Z., Stains J., Ebrat B., Guyonnet D., Legrain-Raspaud S., Trotin B., Naliboff B. (2013). Consumption of Fermented Milk Product with Probiotic Modulates Brain Activity. Gastroenterology.

[7] Kechagia M., Basoulis D., Konstantopoulou S., Dimitriadi D., Gyftopoulou K., Skarmoutsou N., Fakiri E.M. (2013). Health Benefits of Probiotics: A Review. Int. Sch. Res. Not..

[8] Alhaj O.A. (2016). Identification of potential ACE-inhibitory peptides from dromedary fermented camel milk. CyTA J. Food.

[9] Marchesi J.R., Adams D.H., Fava F., Hermes G.D.A., Hirschfield G.M., Hold G.L., Quraishi M.N., Kinross J., Smidt H., Tuohy K.M. (2015). The gut microbiota and host health: A new clinical frontier. Gut.

[10] Turnbaugh P.J., Gordon J.I. (2009). The core gut microbiome, energy balance and obesity. J. Physiol..

[11] Khalesi S., Sun J., Buys N., Jayasinghe R. (2014). Effect of Probiotics on Blood Pressure: A Systematic Review and Meta-Analysis of Randomized, Controlled Trials. Hypertension.

[12] Jose P.A., Raj D. (2015). Gut microbiota in hypertension. Curr. Opin. Nephrol. Hypertens..

[13] Solak Y., Afsar B., Vaziri N.D., Aslan G., Yalcin C.E., Covic A., Kanbay M. (2016). Hypertension as an autoimmune and inflammatory disease. Hypertens. Res..

[14] Abais-Battad J.M., Alsheikh A.J., Pan X., Fehrenbach D.J., Dasinger J.H., Lund H., Roberts M.L., Kriegel A.J., Cowley A.W., Kidambi S. (2019). Dietary Effects on Dahl Salt-Sensitive Hypertension, Renal Damage, and the T Lymphocyte Transcriptome. Hypertension.

[15] Masson G.S., Nair A.R., Soares P.P.S., Michelini L.C., Francis J. (2015). Aerobic training normalizes autonomic dysfunction, HMGB1 content, microglia activation and inflammation in hypothalamic paraventricular nucleus of SHR. Am. J. Physiol. Circ. Physiol..

[16] Sharafetdinov K., Plotnikova O.A., Alexeeva R.I., Sentsova T.B., Songisepp E., Stsepetova J., Smidt I., Mikelsaar M. (2013). Hypocaloric diet supplemented with probiotic cheese improves body mass index and blood pressure indices of obese hypertensive patients—A randomized double-blind placebo-controlled pilot study. Nutr. J..

[17] Naruszewicz M., Johansson M.-L., Zapolska-Downar D., Bukowska H. (2002). Effect of Lactobacillus plantarum 299v on cardiovascular disease risk factors in smokers. Am. J. Clin. Nutr..

[18] Ahrén I.L., Xu J., Önning G., Olsson C., Ahrné S., Molin G. (2015). Antihypertensive activity of blueberries fermented by Lactobacillus plantarum DSM 15313 and effects on the gut microbiota in healthy rats. Clin. Nutr..

[19] Xu J., Ahrén I.L., Prykhodko O., Olsson C., Ahrné S., Molin G. (2013). Intake of Blueberry Fermented byLactobacillus plantarumAffects the Gut Microbiota of L-NAME Treated Rats. Evid.-Based Complement. Altern. Med..

[20] Yang L., Xie X., Li Y., Wu L., Fan C., Liang T., Xi Y., Yang S., Li H., Zhang J. (2021). Evaluation of the Cholesterol-Lowering Mechanism of *Enterococcus faecium* Strain 132 and Lactobacillus paracasei Strain 201 in Hypercholesterolemia Rats. Nutrients.

[21] Tsuchiya K., Tomita S., Ishizawa K., Abe S., Ikeda Y., Kihira Y., Tamaki T. (2010). Dietary nitrite ameliorates renal injury in l-NAME-induced hypertensive rats. Nitric Oxide.

[22] Yildirim F.I.A., Kizilay D.E., Ergin B., Ekmekçi B., Topal G., Kucur M., Tansel C.D., Doğan B.S.U. (2015). Barnidipine ameliorates the vascular and renal injury in l-NAME-induced hypertensive rats. Eur. J. Pharmacol..

[23] Li H., Xie X., Li Y., Chen M., Xue L., Wang J., Zhang J., Wu S., Ye Q., Zhang S. (2021). *Pediococcus pentosaceus* IM96 Exerts Protective Effects against Enterohemorrhagic Escherichia coli O157:H7 Infection In Vivo. Foods.

[24] Liang T., Xie X., Wu L., Li L., Li H., Xi Y., Feng Y., Xue L., Chen M., Chen X. (2021). Microbial Communities and Physiochemical Properties of Four Distinctive Traditionally Fermented Vegetables from North China and Their Influence on Quality and Safety. Foods.

[25] Chen S., Zhou Y., Chen Y., Gu J. (2018). fastp: An ultra-fast all-in-one FASTQ preprocessor. Bioinformatics.

[26] Edgar R.C. (2013). UPARSE: Highly accurate OTU sequences from microbial amplicon reads. Nat. Methods.

[27] Stackebrandt E., Goebel B.M. (1994). Taxonomic Note: A Place for DNA-DNA Reassociation and 16S rRNA Sequence Analysis in the Present Species Definition in Bacteriology. Int. J. Syst. Evol. Microbiol..

[28] Wang Q., Garrity G.M., Tiedje J.M., Cole J.R. (2007). Naive Bayesian classifier for rapid assignment of rRNA sequences into the new bacterial taxonomy. Appl. Environ. Microbiol..

[29] Khan A., Ju F., Xie W., Hafeez M.T., Cheng X., Yang Z., Zhu L., Li T., Zhang S. (2017). Transcriptomic Analysis Reveals Differential Activation of Microglial Genes after Ischemic Stroke in Mice. Neuroscience.

[30] Shalaby S.M., Zakora M., Otte J. (2006). Performance of two commonly used angiotensin-converting enzyme inhibition assays using FA-PGG and HHL as substrates. J. Dairy Res..

[31] Alshuniaber M., Alhaj O., Abdallah Q., Jahrami H. (2021). Effects of camel milk hydrolysate on blood pressure and biochemical parameters in fructose-induced hypertensive rats. Nutr. Food Sci..

[32] Yan X., Jin J., Su X., Yin X., Gao J., Wang X., Zhang S., Bu P., Wang M., Zhang Y. (2020). Intestinal Flora Modulates Blood Pressure by Regulating the Synthesis of Intestinal-Derived Corticosterone in High Salt-Induced Hypertension. Circ. Res..

[33] Yang T., Santisteban M.M., Rodriguez V., Li E., Ahmari N., Carvajal J.M., Zadeh M., Gong M., Qi Y., Zubcevic J. (2015). Gut Dysbiosis Is Linked to Hypertension. Hypertension.

[34] Song T., Guan X., Wang X., Qu S., Chen X. (2021). Dynamic modulation of gut microbiota improves post-yocardial infarct tissue repair in rats via butyric acid-ediated histone deacetylase inhibition. FASEB J..

[35] Strazzera G., Battista F., Andreolli M., Menini M., Bolzonella D., Lampis S. (2021). Influence of different household Food Wastes Fractions on Volatile Fatty Acids production by anaerobic fermentation. Bioresour. Technol..

[36] Wishart D.S., Feunang Y.D., Marcu A., Guo A.C., Liang K., Vázquez-Fresno R., Sajed T., Johnson D., Li C., Karu N. (2018). HMDB 4.0: The human metabolome database for 2018. Nucleic Acids Res..

[37] Chong J., Soufan O., Li C., Caraus I., Li S., Bourque G., Wishart D.S., Xia J. (2018). MetaboAnalyst 4.0: Towards more transparent and integrative metabolomics analysis. Nucleic Acids Res..

[38] Nemet I., Saha P.P., Gupta N., Zhu W., Romano K.A., Skye S.M., Cajka T., Mohan M.L., Li L., Wu Y. (2020). A Cardiovascular Disease-Linked Gut Microbial Metabolite Acts via Adrenergic Receptors. Cell.

[39] Richards J., Diaz A.N., Gumz M.L. (2014). Clock genes in hypertension: Novel insights from rodent models. Blood Press. Monit..

[40] Zhao D., Qi Y., Zheng Z., Wang Y., Zhang X.-Y., Li H.-J., Liu H.-H., Zhang X.-T., Du J., Liu J. (2011). Dietary factors associated with hypertension. Nat. Rev. Cardiol..

[41] Marco M.L., Heeney D., Binda S., Cifelli C.J., Cotter P.D., Foligné B., Gänzle M., Kort R., Pasin G., Pihlanto A. (2017). Health benefits of fermented foods: Microbiota and beyond. Curr. Opin. Biotechnol..

[42] Aguilar A. (2017). Microbiota under pressure. Nat. Rev. Nephrol..

[43] Santisteban M.M., Kim S., Pepine C.J., Raizada M.K. (2016). Brain-Gut-Bone Marrow Axis: Implications for Hypertension and Related Therapeutics. Circ. Res..

[44] Ganesh B., Nelson J.W., Eskew J.R., Ganesan A., Ajami N.J., Petrosino J.F., BryanJr R.M., Durgan D.J. (2018). Prebiotics, Probiotics, and Acetate Supplementation Prevent Hypertension in a Model of Obstructive Sleep Apnea. Hypertension.

[45] Gomez-Guzman M., Toral M., Romero M., Jimenez R., Galindo P., Sanchez M., Zarzuelo M.J., Olivares M., Galvez J., Duarte J. (2015). Antihypertensive effects of probiotics Lactobacillus strains in spontaneously hypertensive rats. Mol. Nutr. Food Res..

[46] Jakala P., Pere E., Lehtinen R., Turpeinen A., Korpela R., Vapaatalo H.J.J.P.P. (2009). Cardiovascular activity of milk casein-derived tripeptides and plant sterols in spontaneously hypertensive rats. J. Physiol. Pharmacol..

[47] Beltrán-Barrientos L.M., García H.S., Hernández-Mendoza A., González-Córdova A.F., Vallejo-Cordoba B.J.J.o.D.S. (2021). Invited review: Effect of antihypertensive fermented milks on gut microbiota. J. Dairy. Sci..

[48] Kong C.-Y., Li Z.-M., Mao Y.-Q., Chen H.-L., Hu W., Han B., Wang L.-S. (2021). Probiotic yogurt blunts the increase of blood pressure in spontaneously hypertensive rats via remodeling of the gut microbiota. Food Funct..

[49] Chen L., Wang L., Li J., Shu G. (2021). Antihypertensive potential of fermented milk: The contribution of lactic acid bacteria proteolysis system and the resultant angiotensin-converting enzyme inhibitory peptide. Food Funct..

[50] Virdis A., Dell’Agnello U., Taddei S.J.M. (2014). Impact of inflammation on vascular disease in hypertension. Maturitas.

[51] Miguel-Carrasco J.L., Mate A., Monserrat M.T., Arias J.L., Aramburu O., Vázquez C. (2008). The role of inflammatory markers in the cardioprotective effect of L-carnitine in L-NAME-induced hypertension. Am. J. Hypertens..

[52] Gonzalez-Gonzalez C.R., Tuohy K.M., Jauregi P.J. (2011). Production of angiotensin-I-converting enzyme (ACE) inhibitory activity in milk fermented with probiotic strains: Effects of calcium, pH and peptides on the ACE-inhibitory activity. Int. Dairy. J..

[53] Yang Z., Zhang M., Jiang Y., Cai M. (2020). Characterization and ACE Inhibitory Activity of Fermented Milk with Probiotic *Lactobacillus plantarum* K25 as Analyzed by GC-MS-Based Metabolomics Approach. J. Microbiol. Biotechnol..

[54] Hao Y., Wang Y., Xi L., Li G., Zhao F., Qi Y., Liu J., Zhao D. (2016). A Nested Case-Control Study of Association between Metabolome and Hypertension Risk. BioMed Res. Int..

[55] Heikal L., Starr A., Hussein D., Prieto-Lloret J., Aaronson P., Dailey L.A., Nandi M. (2018). l-Phenylalanine Restores Vascular Function in Spontaneously Hypertensive Rats Through Activation of the GCH1-GFRP Complex. JACC Basic Transl. Sci..

[56] Wang Z., Cheng C., Yang X., Zhang C. (2021). L-phenylalanine attenuates high salt-induced hypertension in Dahl SS rats through activation of GCH1-BH4. PLoS ONE.

[57] Zhen-Lin L., Ben-Hua Z., Wei W., Gui-Hua L., Fei W., Li W., Qing-Ping Z., Hong W. (2016). Xiang. Impact of the Consumption of Tea Polyphenols on Early Atherosclerotic Lesion Formation and Intestinal Bifidobacteria in High-Fat-Fed ApoE/Mice. Front. Nutr..

[58] Wang J., Gareri C., Rockman H.A. (2018). G-Protein–Coupled Receptors in Heart Disease. Circ. Res..

[59] Robin S., Maupoil V., Laurant P., Jacqueson A., Berthelot A. (2004). Effect of a methionine-supplemented diet on the blood pressure of Sprague–Dawley and deoxycorticosterone acetate–salt hypertensive rats. Br. J. Nutr..

[60] Robin S., Maupoil V., Groubatch F., Laurant P., Jacqueson A., Berthelot A. (2003). Effect of a methionine-supplemented diet on the blood pressure of Wistar–Kyoto and spontaneously hypertensive rats. Br. J. Nutr..

[61] Zhou Y., Zhao L., Zhang Z., Lu X. (2015). Protective Effect of Enalapril against Methionine-Enriched Diet-Induced Hypertension: Role of Endoplasmic Reticulum and Oxidative Stress. BioMed Res. Int..

[62] Mirmiran P., Teymoori F., Asghari G., Azizi F. (2019). Dietary Intakes of Branched Chain Amino Acids and the Incidence of Hypertension: A Population-Based Prospective Cohort Study. Arch. Iran. Med..

[63] Wang X.-J., Gao X., Zhang A.-H., Wu F.-F., Yan G.-L., Sun H. (2019). High-throughput metabolomics for evaluating the efficacy and discovering the metabolic mechanism of Luozhen capsules from the excessive liver-fire syndrome of hypertension. RSC Adv..

[64] Fu Z.D., Cui J.Y. (2017). Remote Sensing Between Liver and Intestine: Importance of Microbial Metabolites. Curr. Pharmacol. Rep..

[65] Hilfenhaus M. (1976). Circadian rhythm of the renin-angiotensin-aldosterone system in the rat. Arch. Toxicol..

[66] Douma L.G., Holzworth M.R., Solocinski K., Masten S.H., Miller A.H., Cheng K.-Y., Lynch I.J., Cain B.D., Wingo C.S., Gumz M.L. (2018). Renal Na-handling defect associated with PER1-dependent nondipping hypertension in male mice. Am. J. Physiol. Physiol..

[67] Tanaka S., Ueno T., Tsunemi A., Nagura C., Tahira K., Fukuda N., Soma M., Abe M. (2018). The adrenal gland circadian clock exhibits a distinct phase advance in spontaneously hypertensive rats. Hypertens. Res..

[68] Feng D.-D., Zheng B., Yu J., Zhang M.-L., Ma Y., Hao X., Wen J.-K., Zhang X.-H. (2021). 17β-Estradiol Inhibits Proliferation and Oxidative Stress in Vascular Smooth Muscle Cells by Upregulating BHLHE40 Expression. Front. Cardiovasc. Med..

