# Supplementary material for: Antihypertensive Activity of Milk Fermented by Lactiplantibacillus plantarum SR37-3 and SR61-2 in L-NAME-Induced Hypertensive Rats
Source: Foods. 2022 Aug 4;11(15):2332. doi: 10.3390/foods11152332 (PMC9367739; doi:10.3390/foods11152332)
Supplement: Supplementary file 1 [file foods-11-02332-s001.zip › foods-1826217-supplementary.pdf]

foods

MDPI

Supplementary Materials

Antihypertensive Activity of Milk Fermented by *Lactiplantibacillus plantarum* SR37-3 and SR61-2 in L-NAME-Induced Hypertensive Rats

Lin Yuan [1,2], Ying Li [2], Moutong Chen [2], Liang Xue [2], Juan Wang [2], Yu Ding [2], Jumei Zhang [2], Shi Wu [2], Qinghua Ye [2], Shuhong Zhang [2], Runshi Yang [2], Hui Zhao [2], Lei Wu [2], Tingting Liang [2], Xinqiang Xie [2] and Qingping Wu [2,*]

Table S1. Detection of different metabolites in serum samples.

Different metabolites	Formula	Molecular Weight	RT [min]	VIP	p-Value	HMDB ID	Scan mode
		Different metabolites between W and LN					
2-palmitoyl-sn-glycero-3-phosphocholine	$C_{24} H_{50} N O_7 P$	495.33	14.12	6.05	0.0282	HMDB0061709	+
L-Valine	$C_5 H_{11} N O_2$	117.08	1.26	5.03	0.0205	HMDB0000883	+
Creatine	$C_4 H_9 N_3 O_2$	131.07	1.02	4.10	0.0301	HMDB0000064	+
Platelet-activating factor	$C_{26} H_{54} N O_7 P$	523.36	17.24	6.47	0.0345	METPA0517	+
L-(-)-Methionine	$C_5 H_{11} N O_2 S$	149.05	1.29	3.62	0.0012	HMDB0000696	+
Crotonic acid	$C_4 H_6 O_2$	86.04	2.30	1.79	0.0311	HMDB0010720	+
Diethylamine	$C_4 H_{11} N$	73.09	25.15	3.72	0.0037	HMDB0041878	+
Acetyl-beta -methylcholine	$C_8 H_{17} N O_2$	159.13	1.25	1.92	0.0280	HMDB0015654	+
5-Methylcytosine	$C_5 H_7 N_3 O$	125.06	1.85	1.05	0.0352	HMDB0002894	+
Oleamide	$C_{18} H_{35} N O$	281.27	21.60	2.48	0.0015	HMDB0002117	+
Phenazone	$C_{11} H_{12} N_2 O$	188.09	3.18	2.67	0.0075	HMDB0015503	+
Nicotinamide	$C_6 H_6 N_2 O$	122.05	1.29	2.61	0.0438	HMDB0001406	+
5-Hydroxyindole-3-acetic acid	$C_{10} H_9 N O_3$	191.06	3.28	2.41	0.0055	HMDB0000763	+
Metixene	$C_{20} H_{23} N S$	309.15	17.24	1.63	0.0362	HMDB0014484	+
4-Hydroxycoumarin	$C_9 H_6 O_3$	162.03	6.52	1.08	0.0331	HMDB0003654	+
Hexylamine	$C_6 H_{15} N$	101.12	25.15	2.01	0.0140	HMDB0032323	+
Betaine	$C_5 H_{11} N O_2$	117.08	25.17	1.22	0.0282	HMDB0000043	+
Palmitoleic Acid	$C_{16} H_{30} O_2$	276.21	15.79	1.04	0.0096	HMDB0003229	+
PEG-4	$C_8 H_{18} O_5$	194.12	3.48	1.09	0.0254	HMDB0061705	+
Glu-leu	$C_{11} H_{20} N_2 O_5$	260.14	4.18	1.11	0.0157	HMDB0028823	+

Noramidopyrine	$C_{12}H_{15}N_3O$	217.12	3.54	1.37	0.0091	HMDB0013839	+
alpha -Linolenic acid	$C_{18}H_{30}O_2$	278.22	20.26	1.18	0.0082	HMDB0001388	+
Histamine	$C_5H_9N_3$	111.08	3.76	1.07	0.0012	HMDB0000870	+
Different metabolites	Formula	Molecular Weight	RT [min]	VIP	p-Value	HMDB ID	Scan mode
Arachidonic acid	$C_{20}H_{32}O_2$	304.24	17.90	6.16	0.0018	HMDB0001043	-
(+/-)11(12)-EET	$C_{20}H_{32}O_3$	320.23	11.67	3.52	0.0277	HMDB0010409	-
8Z,11Z,14Z-Eicosatrienoic acid	$C_{20}H_{34}O_2$	306.26	19.81	1.33	0.0358	HMDB0002925	-
Adrenic acid	$C_{22}H_{36}O_2$	332.27	21.26	1.78	0.0047	HMDB0002226	-
D-(+)-Tryptophan	$C_{11}H_{12}N_2O_2$	204.09	3.76	1.61	0.0010	HMDB0013609	-
beta-Muricholic acid	C24 H40 O5	408.29	7.87	1.19	0.0304	HMDB0000415	-
L-Phenylalanine	C9 H11 N O2	165.08	2.926	1.01	0.0208	HMDB0000159	-
Different metabolites between LN and LN+SR37-3							
L-Phenylalanine	C9 H11 N O2	165.08	3.25	17.64	0.0262	HMDB0000159	+
L-Valine	C5 H11 N O2	117.08	1.25	5.79	0.0130	HMDB0000883	+
Choline	C5 H13 N O	103.10	1.46	1.18	0.0068	HMDB0000097	+
L-(-)-Methionine	C5 H11 N O2 S	149.05	1.29	3.39	0.0174	HMDB0000696	+
Uric acid	C5 H4 N4 O3	168.03	1.72	1.93	0.0175	HMDB0000289	+
Nicotinamide	C6 H6 N2 O	122.05	1.60	1.23	0.0322	HMDB0001406	+
Acetyl-beta-methylcholine	C8 H17 N O2	159.13	1.25	4.42	0.0007	HMDB0015654	+
Phenazone	C11 H12 N2 O	188.09	3.18	3.21	0.0182	HMDB0015503	+
Methyl indole-3-acetate	C11 H11 N O2	189.08	7.87	1.74	0.0416	HMDB0029738	+
Creatinine	C4 H7 N3 O	113.06	0.99	1.39	0.0276	HMDB0000562	+
Diethylamine	C4 H11 N	73.09	25.08	1.42	0.0206	HMDB0041878	+
4-Hydroxycoumarin	C9 H6 O3	162.03	6.52	1.26	0.0055	HMDB0003654	+
Palmitic Acid	C16 H32 O2	273.27	9.27	2.07	0.0319	HMDB0000220	+
Thromboxane B2	C20 H34 O6	352.22	8.28	2.15	0.0041	HMDB0003252	+
muramic acid	C9 H17 N O7	251.10	0.95	1.26	0.0212	HMDB0003254	+
Isoquinoline	C9 H7 N	129.06	7.87	1.23	0.0442	HMDB0034244	+
5-Methylcytosine	C5 H7 N3 O	125.06	1.29	1.25	0.0092	HMDB0002894	+
(+/-)11(12)-EET	C20 H32 O3	302.22	15.48	1.81	0.0007	HMDB0010409	+
Different metabolites	Formula	Molecular Weight	RT [min]	VIP	p-Value	HMDB ID	Scan mode
16-Hydroxyhexadecanoic acid	C16 H32 O3	254.22	16.85	6.85	0.0259	HMDB0006294	-
Gluconic acid	C6 H12 O7	196.06	0.83	1.83	0.0018	HMDB0000625	-
Glycolic acid	C2 H4 O3	76.02	0.84	1.27	0.0276	HMDB0000115	-
Different metabolites between LN and LN+SR61-2							
Platelet-activating factor	C26 H54 N O7 P	523.36	18.40	1.84	0.0032	METPA0517	+
2-palmitoyl-sn-glycero-3-phosphocholine	C24 H50 N O7 P	495.33	14.12	7.22	0.0135	HMDB0061709	+
L-alpha-Glycerylphosphorylcholine	C8 H20 N O6 P	257.10	0.93	5.52	0.0070	HMDB0000086	+

N, N-Dimethylaniline	C8 H11 N	121.09	25.13	4.22	0.0063	HMDB0001020	+
L-Glutamic acid	C5 H9 N O4	147.05	0.94	1.58	0.0341	HMDB0000148	+
3-Aminopropanal	C3 H7 N O	73.05	0.92	2.30	0.0133	HMDB0001106	+
Levetiracetam	C8 H14 N2 O2	170.11	0.92	2.31	0.0121	HMDB0015333	+
L-(-)-Methionine	C5 H11 N O2 S	149.05	1.51	1.70	0.0232	HMDB0000696	+
N-Undecanoylglycine	C13 H25 N O3	243.18	8.51	3.17	0.0311	HMDB0013286	+
Acetyl-beta-methylcholine	C8 H17 N O2	159.13	1.25	3.50	0.0000	HMDB0015654	+
Oleamide	C18 H35 N O	281.27	21.6	1.55	0.0258	HMDB0002117	+
Palmitoylcarnitine	C23 H45 N O4	399.33	14.10	1.47	0.0040	HMDB0000222	+
Acetylcholine	C7 H15 N O2	145.11	1.04	1.46	0.0131	HMDB0000895	+
5-Hydroxyindole-3-acetic acid	C10 H9 N O3	191.06	3.28	1.96	0.0123	HMDB0000763	+
Metixene	C20 H23 N S	309.15	17.24	1.60	0.0032	HMDB0014484	+
Arachidonic acid	C20 H32 O2	304.24	16.86	1.63	0.0443	HMDB0001043	+
Diethylamine	C4 H11 N	73.09	25.08	1.12	0.0266	HMDB0041878	+
Betaine	C5 H11 N O2	117.08	25.17	1.07	0.0333	HMDB0000043	+
5-Methylcytosine	C5 H7 N3 O	125.06	1.29	1.23	0.0001	HMDB0002894	+
Dihydrothymine	C5 H8 N2 O2	128.06	1.01	1.18	0.0016	HMDB0000079	+
Spinacine	C7 H9 N3 O2	167.07	0.80	1.06	0.0024	HMDB0029873	+
Linoleic acid	C18 H32 O2	280.24	18.28	6.07	0.0418	HMDB0000673	-
Different metabolites	Formula	Molecular Weight	RT [min]	VIP	p-Value	HMDB ID	Scan mode
Elaidic acid	C18 H34 O2	282.26	21.04	6.15	0.0031	HMDB0000573	-
Arachidonic acid	C20 H32 O2	304.24	17.90	2.99	0.0415	HMDB0001043	-
Stearic acid	C18 H36 O2	284.27	23.28	6.42	0.0290	HMDB0000827	-
Adrenic acid	C22 H36 O2	332.27	21.26	1.03	0.0104	HMDB0002226	-
Gluconic acid	C6 H12 O7	196.06	0.83	1.29	0.0274	HMDB0000625	-
Erythrose	C4 H8 O4	120.04	0.83	1.08	0.0098	HMDB0002649	-

R.T.: retention time (minute), cont W: animals not treated with L-NAME receiving standard chow, cont LN: L-NAME treated rats receiving standard chow, LN+SR37-3: L-NAME treated rats receiving PFM-SR37-3, LN+SR61-2: L-NAME treated rats receiving PFM-SR61-2. VIP: variable importance projection. HMDB: The Human Metabolome Database.

Table S2. Detection of different metabolites in cecal contents samples.

Different metabolites	Formula	Molecular Weight	RT [min]	VIP	p-Value	HMDB ID	Scan mode
Different metabolites between W and LN							
Oleamide	C18 H35 N O	281.27	20.80	27.14	0.0000	HMDB0002117	+
Hexadecanamide	C16 H33 N O	255.26	19.72	12.63	0.0000	HMDB0012273	+
Linoleamide	C18 H33 N O	279.26	18.53	13.22	0.0000	HMDB0062656	+
Stearamide	C18 H37 N O	283.29	24.72	8.77	0.0031	HMDB0034146	+
3b-Hydroxy-5-cholenoic acid	C24 H38 O3	374.29	11.32	8.79	0.0002	HMDB0000308	+
L-Phenylalanine	C9 H11 N O2	165.08	2.21	9.72	0.0000	HMDB0000159	+
L-Norleucine	C6 H13 N O2	131.09	1.55	9.73	0.0000	HMDB0001645	+
3a,7a-Dihydroxycholanoic acid	C24 H40 O4	392.29	11.32	5.93	0.0016	HMDB0000384	+
2-Pyrrolidone	C4 H7 N O	85.05	1.22	7.68	0.0000	HMDB0002039	+
L-Valine	C5 H11 N O2	117.08	1.07	7.13	0.0000	HMDB0000883	+
Tiglic acid	C5 H8 O2	100.05	1.07	7.12	0.0000	HMDB0001470	+
Ornithine	C5 H12 N2 O2	132.09	0.80	7.01	0.0001	HMDB0000214	+
N-Acetyl-b-D-glucosamine	C8 H15 N O6	221.09	0.93	3.47	0.0028	HMDB0000803	+
2b,3a,7a-Trihydroxy-5b-cholanoic acid	C24 H40 O5	408.29	8.88	3.28	0.0025	HMDB0000404	+
Ricinoleic Acid	C18 H34 O3	298.25	14.08	4.44	0.0035	HMDB0034297	+
L-(-)-Methionine	C5 H11 N O2 S	149.05	1.26	2.97	0.0020	HMDB0000696	+
Valine	C5 H11 N O2	117.08	1.35	2.79	0.0000	HMDB0000883	+
DL-Tryptophan	C11 H12 N2 O2	204.09	3.67	4.23	0.0000	HMDB0013609	+
Gly-Phe	C11 H14 N2 O3	222.10	3.61	2.67	0.0202	HMDB0028848	+
chenodeoxycholic acid	C24 H40 O4	392.29	8.57	4.14	0.0000	HMDB0000518	+
Furfuranol	C5 H6 O2	98.037	1.23	2.17	0.0239	HMDB0013742	+
3-(3,4-dihydroxyphenyl) propanoic acid	C9 H10 O4	164.05	1.18	3.22	0.0000	HMDB0000423	+
L-Threonine	C4 H9 N O3	87.03	1.06	2.24	0.0146	HMDB0000167	+
Adenine	C5 H5 N5	135.05	1.00	2.03	0.0022	HMDB0000034	+
Different metabolites	Formula	Molecular Weight	RT [min]	VIP	p-Value	HMDB ID	Scan mode
Deoxycholic Acid	C24 H40 O4	392.29	7.79	13.58	0.0110	HMDB0000626	-
Oleic Acid	C18 H34 O2	282.26	19.54	4.92	0.0269	HMDB0000207	-
7-ketodeoxycholic acid	C24 H38 O5	406.27	5.95	2.18	0.0000	HMDB0000391	-
Different metabolites between LN and LN+SR37-3							
3b-Hydroxy-5-cholenoic acid	C24 H38 O3	374.28	11.32	12.71	0.0426	HMDB0000308	+
N-Acetyl-b-D-glucosamine	C8 H15 N O6	221.09	0.93	12.64	0.0002	HMDB0000803	+
Uracil	C4 H4 N2 O2	95.00	1.16	4.34	0.0322	HMDB0000300	+
Hypoxanthine	C5 H4 N4 O	136.04	1.28	5.34	0.0135	HMDB0000157	+
Thymine	C5 H6 N2 O2	126.04	1.66	5.23	0.0067	HMDB0000262	+
6-Methylquinoline	C10 H9 N	143.07	4.00	4.62	0.0457	HMDB0033115	+
Adenine	C5 H5 N5	135.05	1.00	6.49	0.0016	HMDB0000034	+

L-Valine	C5 H11 N O2	117.08	0.96	4.69	0.0474	HMDB0000883	+
Xanthine	C5 H4 N4 O2	152.03	1.30	3.85	0.0048	HMDB0000292	+
2-Oxindole	C8 H7 N O	133.05	5.14	4.52	0.0207	HMDB0062549	+
Urocanic acid	C6 H6 N2 O2	138.04	1.06	2.89	0.0225	HMDB0000301	+
Nicotinic acid	C6 H5 N O2	123.03	1.16	3.35	0.0171	HMDB0001488	+
ACPC	C4 H7 N O2	101.05	0.97	4.22	0.0042	HMDB0000230	+
Formiminoglutamic Acid	C6 H10 N2 O4	174.06	0.92	5.65	0.0110	HMDB0000854	+
2'-Deoxyinosine	C10 H12 N4 O4	252.09	1.18	4.34	0.0104	HMDB0000071	+
FF-MAS	C29 H46 O	410.35	19.89	3.16	0.0252	HMDB0001023	+
Acetyl-beta-methylcholine	C8 H17 N O2	159.13	1.03	3.30	0.0186	HMDB0015654	+
Levulinic acid	C5 H8 O3	116.05	1.01	2.33	0.0220	HMDB0000720	+
5-(Hydroxymethyl)-2-furaldehyde	C6 H6 O3	126.03	0.97	2.39	0.0247	HMDB0034355	+
Glycyl-L-leucine	C8 H16 N2 O3	188.12	2.78	2.52	0.0192	HMDB0028929	+
THC	C21 H30 O2	314.22	14.85	2.23	0.0370	HMDB0014613	+
Lotaustralin	C11 H19 N O6	261.12	1.07	2.92	0.0053	HMDB0033865	+

Different metabolites	Formula	Molecular Weight	RT [min]	VIP	p-Value	HMDB ID	Scan mode
Butobarbital	C10 H16 N2 O3	212.12	1.18	2.6	0.0030	HMDB0015442	+
Glutarylcarnitine	C12 H21 N O6	275.14	1.18	2.49	0.0014	HMDB0013130	+
2,3,4,5-tetrahydrodipicolinic acid	C7 H9 N O4	171.05	1.20	2.42	0.0058	HMDB0012289	+
Pyridoxamine	C8 H12 N2 O2	168.09	0.83	2.26	0.0011	HMDB0001431	+
Phthalic acid	C8 H6 O4	166.03	7.34	2.71	0.0000	HMDB0002107	+
Sulfacytine	C12 H14 N4 O3 S	294.08	1.18	2.26	0.0006	HMDB0015412	+
3-(2-Hydroxyethyl) indole	C10 H11 N O	161.08	5.96	2.19	0.0011	HMDB0003447	+
Alosetron	C17 H18 N4 O	294.15	5.05	2.01	0.0001	HMDB0015104	-
16-ketoestrone	C18 H20 O3	284.14	5.56	2.17	0.0000	HMDB0000372	-
Different metabolites between LN and LN+SR61-2							
3b-Hydroxy-5-cholenoic acid	C24 H38 O3	374.28	11.32	15.86	0.0295	HMDB0000308	+
L-Norleucine	C6 H13 N O2	131.09	1.55	13.99	0.0298	HMDB0001645	+
N-Acetyl-b-D-glucosamine	C8 H15 N O6	221.09	0.93	11.27	0.0450	HMDB0000803	+
Ricinoleic Acid	C18 H34 O3	298.25	14.08	10.30	0.0394	HMDB0034297	+
3a,7b,12b-Trihydroxy-5b-cholanoic acid	C24 H40 O5	408.29	7.10	8.67	0.0177	HMDB0000390	+
Hypoxanthine	C5 H4 N4 O	136.04	1.17	5.92	0.0103	HMDB0000157	+
Thymine	C5 H6 N2 O2	126.04	1.66	3.65	0.0274	HMDB0000262	+
Phthalic acid	C8 H6 O4	166.03	12.42	2.11	0.0001	HMDB0002107	+
Glycocholic acid	C26 H43 N O6	465.31	7.07	4.67	0.0032	HMDB0000138	+
THC	C21 H30 O2	314.22	6.96	2.37	0.0199	HMDB0014613	+
Butobarbital	C10 H16 N2 O3	212.12	1.18	2.30	0.0418	HMDB0015442	+
Furfuranol	C5 H6 O2	98.04	1.00	2.18	0.0455	HMDB0013742	+
3-Oxocholic acid	C24 H38 O5	406.27	5.84	2.07	0.0062	HMDB0000502	+
Glycochenodeoxycholic acid	C26 H43 N O5	449.31	8.42	2.37	0.0146	HMDB0000637	+

Lithocholic acid	C24 H40 O3	358.29	14.42	2.18	0.0118	HMDB0000761	+
Dulxanthone H	C22 H22 O8	414.13	6.92	2.22	0.0471	HMDB0034936	+
Different metabolites	Formula	Molecular Weight	RT [min]	VIP	p-Value	HMDB ID	Scan mode
Delta-Valerolactam	C5 H9 N O	116.1	0.90	2.23	0.0029	HMDB0011749	+
1-Hydroxy-3-octanone	C8 H16 O2	144.12	7.89	2.36	0.0000	HMDB0031290	+
Deoxycholic Acid	C24 H40 O4	392.29	7.79	18.92	0.0045	HMDB0000626	-
Lithocholic Acid	C24 H40 O3	376.3	10.68	9.19	0.0487	HMDB0000761	-
Varanic acid	C26 H44 O5	436.32	10.6	6.27	0.0392	HMDB0002195	-
7-ketodeoxycholic acid	C24 H38 O5	406.27	5.95	2.44	0.0255	HMDB0000391	-
10-GINGEROL	C21 H34 O4	350.25	6.92	2.05	0.0263	HMDB0005783	-
Alosetron	C17 H18 N4 O	294.15	5.05	2.96	0.0001	HMDB0015104	-

R.T.: retention time (minute), cont W: animals not treated with L-NAME receiving standard chow, cont LN: L-NAME treated rats receiving standard chow, LN+SR37-3: L-NAME treated rats receiving PFM-SR37-3, LN+SR61-2: L-NAME treated rats receiving PFM-SR61-2. VIP: variable importance projection. HMDB: The Human Metabolome Database.
